# Microbial cells can cooperate to resist high-level chronic ionizing radiation

**DOI:** 10.1371/journal.pone.0189261

**Published:** 2017-12-20

**Authors:** Igor Shuryak, Vera Y. Matrosova, Elena K. Gaidamakova, Rok Tkavc, Olga Grichenko, Polina Klimenkova, Robert P. Volpe, Michael J. Daly

**Affiliations:** 1 Center for Radiological Research, Columbia University, New York, NY, United States of America; 2 Uniformed Services University of the Health Sciences, School of Medicine, Bethesda, MD, United States of America; 3 Henry M. Jackson Foundation for the Advancement of Military Medicine, Bethesda, MD, United States of America; University of Arkansas for Medical Sciences College of Pharmacy, UNITED STATES

## Abstract

Understanding chronic ionizing radiation (CIR) effects is of utmost importance to protecting human health and the environment. Diverse bacteria and fungi inhabiting extremely radioactive waste and disaster sites (*e*.*g*. Hanford, Chernobyl, Fukushima) represent new targets of CIR research. We show that many microorganisms can grow under intense gamma-CIR dose rates of 13–126 Gy/h, with fungi identified as a particularly CIR-resistant group of eukaryotes: among 145 phylogenetically diverse strains tested, 78 grew under 36 Gy/h. Importantly, we demonstrate that CIR resistance can depend on cell concentration and that certain resistant microbial cells protect their neighbors (not only conspecifics, but even radiosensitive species from a different phylum), from high-level CIR. We apply a mechanistically-motivated mathematical model of CIR effects, based on accumulation/removal kinetics of reactive oxygen species (ROS) and antioxidants, in bacteria (3 *Escherichia coli* strains and *Deinococcus radiodurans*) and in fungi (*Candida parapsilosis*, *Kazachstania exigua*, *Pichia kudriavzevii*, *Rhodotorula lysinophila*, *Saccharomyces cerevisiae*, and *Trichosporon mucoides*). We also show that correlations between responses to CIR and acute ionizing radiation (AIR) among studied microorganisms are weak. For example, in *D*. *radiodurans*, the best molecular correlate for CIR resistance is the antioxidant enzyme catalase, which is dispensable for AIR resistance; and numerous CIR-resistant fungi are not AIR-resistant. Our experimental findings and quantitative modeling thus demonstrate the importance of investigating CIR responses directly, rather than extrapolating from AIR. Protection of radiosensitive cell-types by radioresistant ones under high-level CIR is a potentially important new tool for bioremediation of radioactive sites and development of CIR-resistant microbiota as radioprotectors.

## Introduction

Acute ionizing radiation (AIR) is a standard tool for radiobiological experiments. Its use consists of delivering the total ionizing radiation (IR) dose to cells over a time that is too short for substantial damage repair to occur (*e*.*g*. <5 minutes), and/or delivering IR under non-physiological conditions (*e*.*g*. at low temperature) which slow down or stop such repair and genome replication. In contrast, chronic ionizing radiation (CIR) involves continuous or intermittent exposure to IR over an extended time (*e*.*g*. multiple cell generation times), generally under physiological conditions where cells are metabolically active and can proliferate.

Understanding CIR effects on biological systems is important for dealing with the consequences of occupational/medical exposures (nuclear industry workers, astronauts, radiotherapy patients), terrorist attacks involving radioactive materials, nuclear power plant accidents (Chernobyl, Fukushima), and often overlooked, the hazards of Cold War radioactive waste sites, including the Hanford facility [1,2]. Moreover, after decades of advances in space technology and propulsion, CIR in space has remained the most intractable, most severe, obstacle to planning manned Mars missions [3,4]. Despite such broad relevance of CIR, this topic has been under-studied compared with AIR. This occurs mainly because of technical constraints and stringent security measures that limit experimental design and long-term access to low dose rate irradiation facilities.

Highly radioactive waste sites contain diverse microbial inhabitants, including bacteria and fungi. Surprisingly, numerous AIR-sensitive bacteria (*e*.*g*. *Microbacterium*, *Nocardia*, *Pseudomonas*) [1,5] were isolated from highly radioactive sediments at the Hanford facility together with extremely IR-resistant species (*e*.*g*. *Deinococcus radiodurans*, DR) [1]. This finding suggested some form of cellular cooperation under CIR between IR-resistant and IR-sensitive microorganisms, but the idea was dismissed at the time due to lack of experimental evidence.

Survival following exposure to AIR can be facilitated by cell division delays which allow radiogenic damage to be repaired before cell replication [6–8]. Under CIR, however, damage induction is relentless, and an excessively prolonged cell division delay leads to cell death. We therefore hypothesized that qualitative differences exist between physiological factors needed to counteract AIR and CIR stresses: AIR resistance requires coping with massive *amounts* of accumulated radiogenic damage, whereas CIR resistance requires rapid *rates* of damage repair to counteract continuous damage production.

Reactive oxygen species (ROS) are important contributors to IR-induced cell damage and are counteracted by antioxidants, as well as by cell concentration-dependent defenses and by intercellular communication [9–13]. ROS-mediated oxidative stress imposed by AIR is transient, whereas oxidative stress imposed by CIR is, by definition, chronic and persistent. We therefore reasoned that dealing with ROS-mediated damage by intracellular and extracellular mechanisms may be more important for CIR resistance than for AIR resistance.

We tested these hypotheses by measuring and analyzing AIR and CIR responses in multiple phylogenetically diverse fungi and bacteria. Specifically, in one series of experiments we determined resistance to AIR (the dose required to kill 90% of the cells, D_10_) and resistance to CIR (ability to grow under 36 Gy/h) in the same growth medium in 145 fungal strains. In another series of experiments, we investigated CIR resistance in detail in 10 selected microorganisms (4 bacteria and 6 fungi) by exposing them to different CIR dose rates (13–180 Gy/h) at different initial cell concentrations (varied over 5 orders of magnitude). Within our experimental framework, we formulated and tested a mechanistically motivated mathematical model of CIR effects, which explained an organism’s growth-inhibitory CIR critical dose rate by quantifying the impact of cell concentration on ROS/antioxidant production/removal rates.

## Results

### Growth of bacteria and fungi under CIR

**Fig 1:**
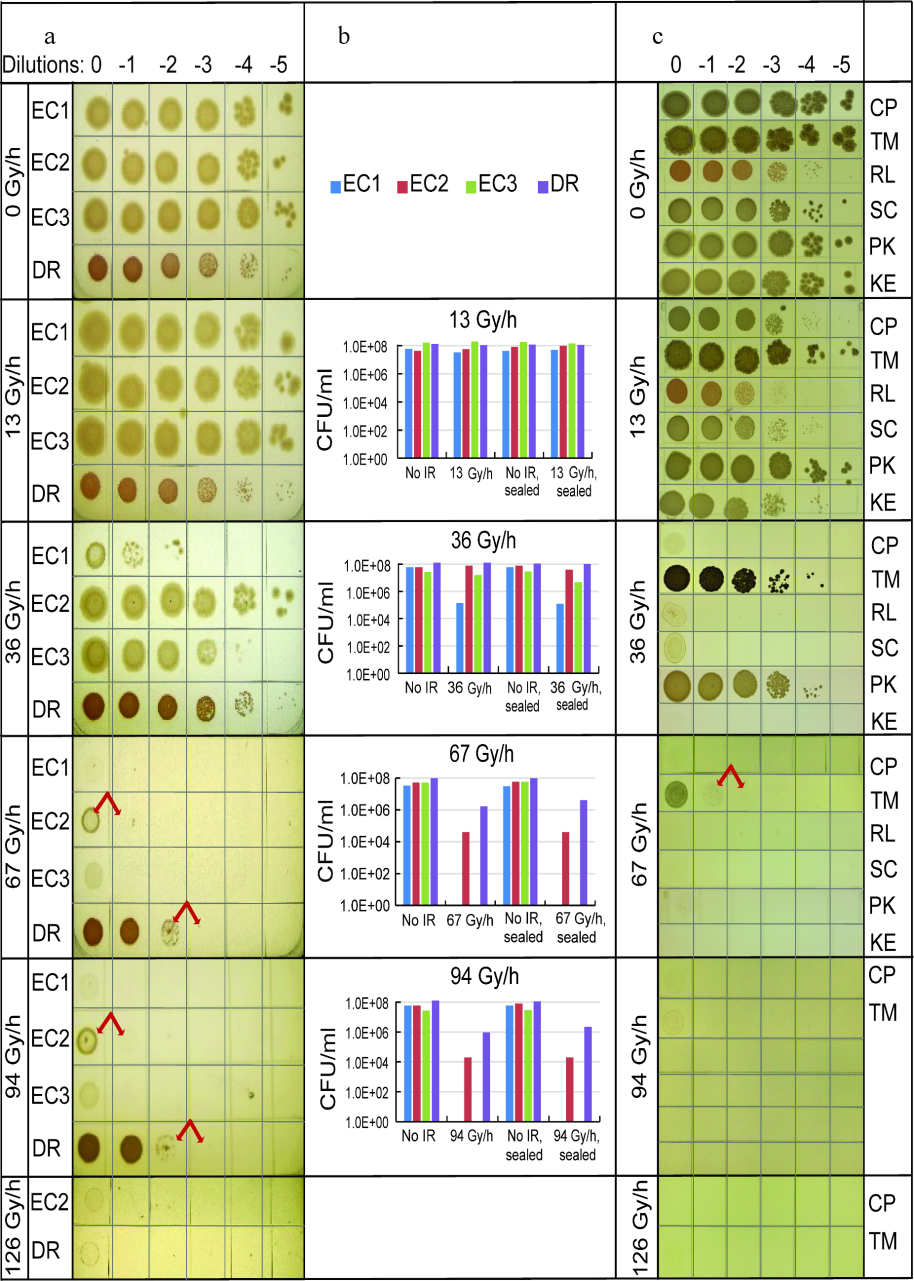
Aerobic growth of microorganisms under CIR. **a:** Bacteria. **b:** Clonogenic survival of bacteria under CIR. For the corresponding CIR study under microaerobic conditions, see S1 Fig. In this and the following figure, dilutions shown in panels **a** and **c** are on a log_10_ scale and represent order of magnitude changes in initial cell concentration. The bars shown in panel **b** are based on CFU counts normalized to 1 ml: the actual numbers of viable cells are 200 times smaller because only 5 μl of each species were used in these experiments. At 94 Gy/h, individual colonies could not always be reliably identified, and therefore the bars at this dose rate represent estimates. Abbreviations: No IR = no irradiation; sealed = microaerobic. Red arrows indicate cases where 10-fold reduction in cell concentration completely extinguished growth at a given dose rate. **c:** Fungi.

The growth of those bacteria (3 *Escherichia coli* strains, abbreviated as EC1, EC2 and EC3, and *Deinococcus radiodurans*, DR) and fungi (*Candida parapsilosis* CP, *Kazachstania exigua* KE, *Pichia kudriavzevii* PK, *Rhodotorula lysinophila* RL, *Saccharomyces cerevisiae* SC, and *Trichosporon mucoides* TM), which was investigated in detail under different CIR dose rates, is shown in **Fig 1** and **S1A Fig**. At each tested dose rate, six sequential log_10_ dilutions (labeled 0, -1, -2, -3, -4 and -5) of cell-containing suspensions were plated onto solid media immediately before irradiation began. These inocula contained approximately 10^6^, 10^5^, 10^4^, 10^3^, 10^2^, and 10^1^ cells, respectively.

Among the microorganisms tested in this manner, the most CIR-resistant were DR, EC2 and TM (**Fig 1, S1A Fig**). At the highest tested cell concentrations (0 dilution, ~10^6^ plated cells) under aerobic conditions (unrestricted air access to growing cultures), these organisms could grow under 126, 94, and 67 Gy/h, respectively. Microaerobic conditions, generated by restricting air access by parafilm covering, enhanced bacterial growth at the highest dose rates yielding discernable growth but did not increase the growth-inhibitory critical dose rates (**S1A Fig**). Irradiated cells were also allowed to recover without CIR (**S1B and S1C Fig**), and clonogenic survival of these post-CIR cultures confirmed the ranking of CIR resistance: DR>EC2>TM (**Fig 1**).

EC2 and EC3 mutants were originally selected from wild-type EC1 by directed evolution, which consisted of the successive passage of EC1 cells through fractionated AIR exposures lethal to most cells [14]. The CIR resistance of these AIR-resistant mutants was not previously tested. We found that the highest dose rate supporting growth at high cell concentrations was 36 Gy/h for EC1, but 94 Gy/h for EC2 (**Fig 1A**). EC3 was considerably more CIR-resistant than the founder EC1, but not as resistant as EC2 (**Fig 1A and 1B**). This is a good example of how EC mutations, which probably elicit only subtle changes in cellular physiology, can result in major enhancements of CIR resistance. In addition, we have shown before that EC can be made very CIR-resistant by simply enriching its growth medium with Mn^2+^ and orthophosphate, which spontaneously form potent Mn-antioxidant complexes [15,16].

Importantly, the ability of several tested organisms to withstand a given dose rate was strongly dependent on the initial cell concentration. For example, at 67 Gy/h DR grew robustly at high cell concentrations (dilutions 0, -1, -2). However, when the cell concentration decreased 10-fold (from dilution -2 to -3), DR growth at the same dose rate was extinguished (**Fig 1A**). The same pattern occurred at 94 Gy/h. At -2 dilution at least 100 cells remained clonogenically viable under continuous irradiation because, in our experience, 100 is the smallest number of colonies needed to form a uniform lawn covering the inoculated area (**Fig 1A**). At the next (-3) dilution, one would expect 10-fold fewer viable cells resulting in ~10 colonies, but none were seen (**Fig 1A**). This finding is very unlikely to have occurred by chance: if each cell had an equal probability of surviving, independent of other cells, the probability to observe 0 colonies where the expected (mean) number is 10 would be only 1.25×10^−11^ according to the Poisson distribution. A much more plausible explanation is that the growth-inhibitory critical dose rate for DR increased markedly, ~3-fold, with increasing cell density: from somewhere between 36 and 67 Gy/h at -5 dilution to between 126 and 180 Gy/h at 0 dilution. Other bacteria and fungi (*e*.*g*. EC2, TM) could also proliferate under higher CIR dose rates at high cell densities, than as single cells (**Fig 1A–1C**).

Molecular oxygen (O_2_) exacerbates IR toxicity. This phenomenon is named the oxygen effect [17,18]. In our experiments under aerobic conditions, the rate of oxygen diffusion from air into the microbial culture should counteract the rate of oxygen consumption by the small numbers of cells (~10^1^ to ~10^6^) deposited on the surface of agar plates with atmosphere-accessible lids. Also, the per cell oxygen consumption rates of closely related EC1 and EC2 strains are likely to be similar, but the dependences of CIR resistance on cell concentration for these strains were quite different: a marked increase in EC2 resistance occurred between -1 and 0 dilutions, whereas no change occurred at these same dilutions for EC1 (**Fig 1A**). Consequently, radioprotection by O_2_ depletion is unlikely, under our experimental conditions, to explain the observed dependence of CIR resistance on cell concentration in several tested microorganisms (**Fig 1A**).

Instead, the dependence (sometimes very strong, *e*.*g*. for DR at 67 and 94 Gy/h) of CIR resistance on the number of initially plated cells most likely results from interactions between cells. Cell-cell signaling may be involved, but we argue that the simplest plausible explanation is consistent with our model assumptions: because cells can collectively detoxify radiogenic ROS, the cell inoculum survives and grows under CIR only if it initially contains a sufficiently large number of cells to control ROS buildup in the growth medium. By comparison, radiogenic ROS can easily overwhelm inocula that contain only a few cells.

**Fig 2:**
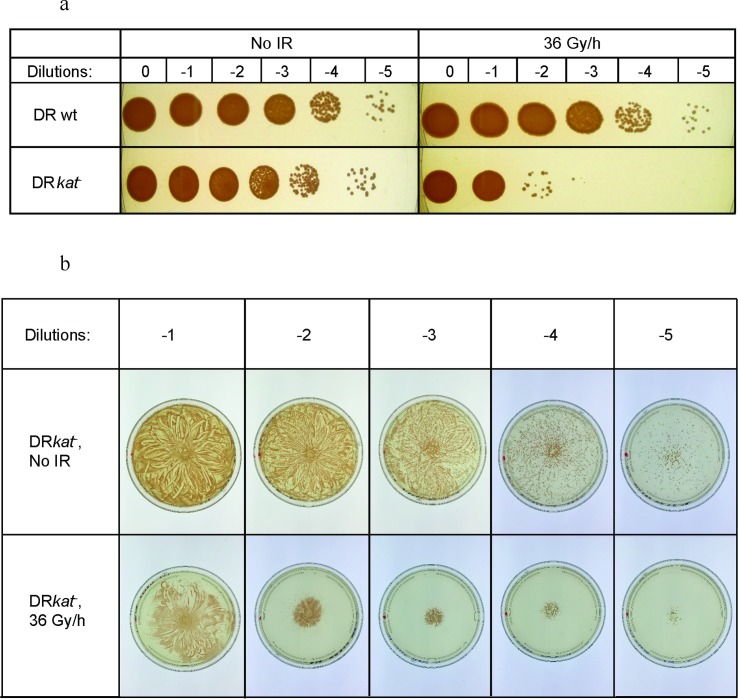
Effect of catalase on CIR resistance. **a**: Growth of DR and DR*kat*^-^ under 36 Gy/h, or without CIR. Dilutions of DR and DR*kat*^-^ are indicated. **b**: Growth restoration of DR*kat*^-^ under 36 Gy/h by catalase, added to the central area of a TGY plate that was pre-inoculated with DR*kat*^-^ cells. Dilutions (log_10_ based) of inoculated DR*kat*^-^ are indicated.

The importance of ROS detoxification for proliferation under CIR was further tested in experiments with a catalase-A-negative *D*. *radiodurans* mutant called DR*kat*^-^ [19]. DR*kat*^-^ was much more sensitive to CIR than the wild-type (**Fig 2A**), and exogenous purified catalase restored its CIR resistance to wild-type levels (**Fig 2B**). Notably, the catalase gene (DR1998) is not required for wild-type levels of AIR resistance in DR [19]. This is consistent with our model assumption that the ROS detoxification rate (i.e. antioxidant status) is more important under CIR than AIR [20,21].

### Insights from mathematical modeling

**Fig 3:**
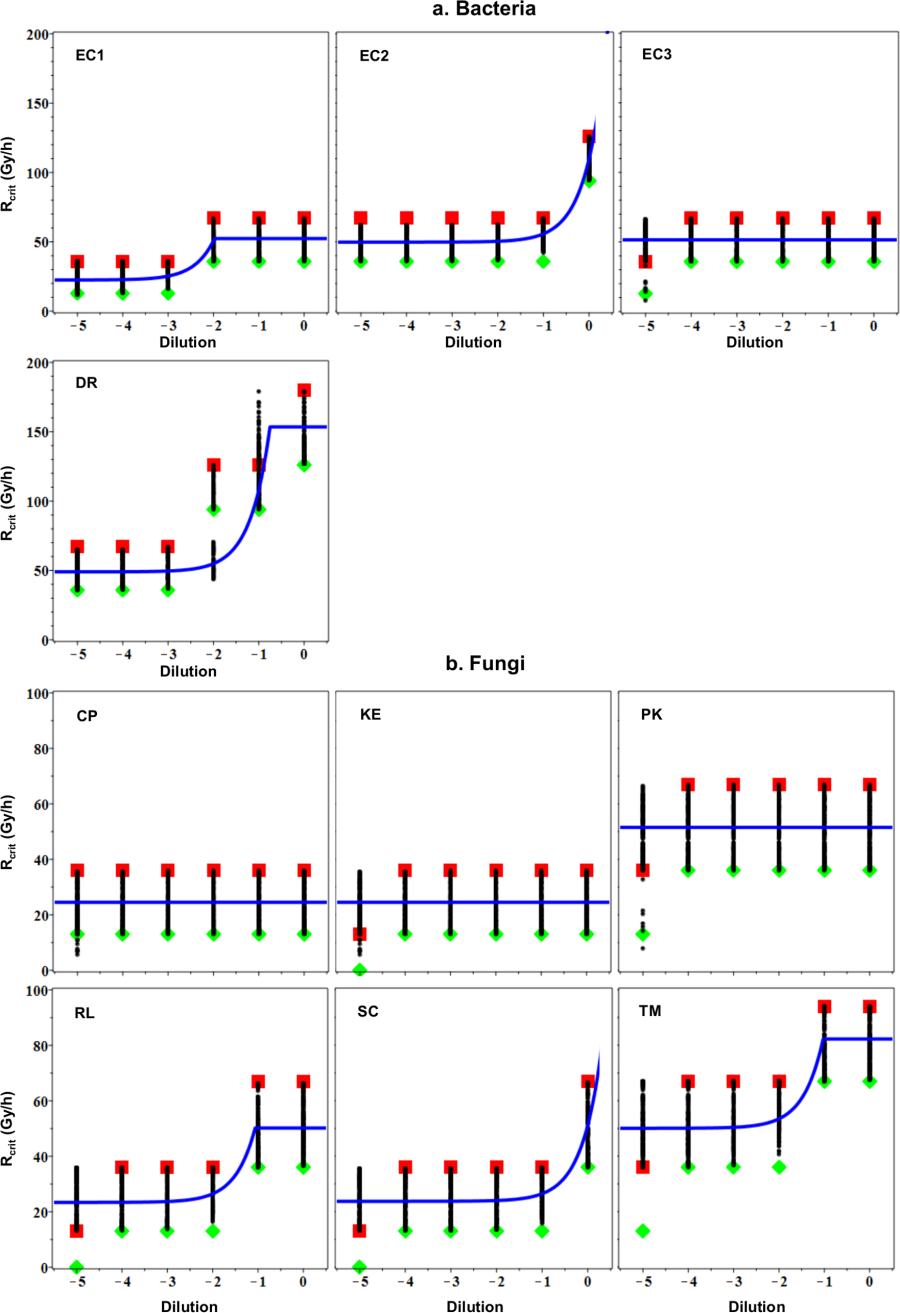
Comparison of observed and model-predicted growth-inhibitory critical CIR dose rates for microorganisms grown under aerobic conditions. **a**: Bacteria. **b**: Fungi. Green diamonds: *highest* tested dose rate at which *any* growth was observed. Red squares: *lowest* tested dose rate at which *no* growth was observed. Blue curves: best-fit model predictions. Black points: uncertainty range of model predictions. Model-based predictions at cell concentrations higher than those tested had very large uncertainties for EC2 and SC and, therefore, the prediction curves were truncated at cell concentrations slightly above 0 dilution for these organisms.

Our mathematical model, despite its simplicity, captured the main data patterns (**Fig 3**): best-fit predicted growth-inhibitory critical dose rates (Eq 7, Materials and Methods, Mechanistic mathematical model) were consistent with observed values for all microorganisms for which the effects of cell concentration on CIR resistance were tested in detail. Discrepancies between predictions and data occurred mostly at the lowest cell concentration (-5 dilution), where the reliability of a deterministic model is limited due to possible stochastic extinction of all of the few plated cells (~10 cells).

The model quantified evidence of cooperative cell radioprotection through *extra*cellular mechanisms by the *slope* parameter in Eq 8B (Materials and Methods, Mechanistic mathematical model). For simplicity, the slope calculation used arbitrary cell concentration units c*: the cell concentration was assumed to be 10^*dil*^, where *dil* is the log_10_ serial dilution (from 0 to -5). For example, at *dil* = 0, c* = 1, whereas at *dil* = -3, c* = 0.001. The *intercept* parameter (Eq 8A, Materials and Methods, Mechanistic mathematical model) provided information about *intra*cellular antioxidant capacities, with units of dose rate (Gy/h). The contribution of DNA double strand breaks (DSBs) to limiting cell proliferation under CIR was represented by parameter R_critDSB_ (Eq 6, Materials and Methods, Mechanistic mathematical model), also with units of dose rate.

The uncertainties in parameter estimates for different organisms were quite large. This occurred because many different intercept/slope/R_critDSB_ combinations (*e*.*g*. small intercept and large slope, or vice versa) produced predictions consistent with the data since the intervals between low and high limits of observed growth-inhibitory critical dose rates were wide (*e*.*g*. 36–67 Gy/h). In other words, many model fit lines with different slope and intercept values could pass through most/all of the critical dose rate intervals for a given microorganism. Predictions from these numerous acceptable fits are shown as black points in **Fig 3**. In most cases, these points span nearly the entire width of the critical dose rate intervals and visually appear as vertical “bars”.

Due to these large uncertainties, it is more informative to compare the lower 95% confidence interval (CI) bounds, rather than point estimates, for the model parameters (**Table 1**). These bounds for the *slope* provide an indication of whether there is evidence for *cooperative radioprotection* (a positive lower 95% CI bound) or not (a negative lower 95% CI bound). In contrast, lower 95% CI bounds for the *intercept* indicate the growth-inhibitory critical dose rate for *individual cells* of the given organism when these cells are plated at low concentration and, therefore, cannot assist each other in coping with CIR. Finally, lower *R*_*critDSB*_ CI bounds provide information about the speed/efficiency of *DSB repair* pathways.

**Table 1:** Confidence intervals (95% CIs) for best-fit model parameter values.

Species (abbreviation), kingdom	Strain	Intercept, Gy/h		Slope, ×100 Gy/(h×c*)		R_critDSB_, Gy/h	
*D*. *radiodurans* (DR), bacteria	ATCC BAA-816	**36.3**	65.2	**3.58**	80.02	**127.1**	175.2
*E*. *coli* (EC1), bacteria	K-12 MG1655 CF1648	**12.2**	35.6	**1.10**	195.53	**36.5**	66.3
*E*. *coli* (EC2), bacteria	CB1000, [14]	**36.5**	62.0	**0.37**	2.03	**96.8**	>200
*Rhodotorula lysinophila* (RL), fungi	EXF-1534	**13.3**	35.7	**0.07**	17.39	**36.7**	>200
*Saccharomyces cerevisiae* (SC), fungi	EXF-5294	**13.4**	35.2	**0.02**	1.26	**38.0**	>200
*Trichosporon mucoides* (TM), fungi	EXF-1444	**36.2**	66.8	**0.05**	20.23	**67.9**	>200

The intercept and slope parameters were mathematically defined in Eqs 8A and 8B, and R_critDSB_ in Eq 6 (Materials and Methods, Mechanistic mathematical model). Briefly, the intercept is related to *intra*cellular antioxidant capacity, the slope is related to *extra*cellular antioxidant capacity, and R_critDSB_ is related to DNA repair. Parameter values are not shown for EC3 (CB2000), *Candida parapsilosis* (CP, EXF-517), *Kazachstania exigua* (KE, EXF-6402), and *Pichia kudriavzevii* (PK, EXF-6398) because no distinct solutions could be determined for these organisms which exhibited no change in growth-inhibitory critical dose rate with cell concentration, as described in the main text, Results section. CIs = Confidence intervals. c* = Arbitrary cell concentration units. The cell concentration in these calculations was assumed to be 10^*dil*^, where *dil* is the log_10_ serial dilution (from 0 to -5). For example, at *dil* = 0, c* = 1, whereas at *dil* = -3, c* = 0.001.

Among the 10 tested microorganisms, our model-based analysis was most informative for those 6 (DR, EC1, EC2, RL, SC, TM) which showed changes in growth-inhibitory critical dose rate with cell concentration. The lower 95% CI slope bounds were positive in these 6 organisms and highest in DR (**Table 1**). The lower 95% CI bounds for the intercept and for R_critDSB_ were highest in DR, EC2 and TM (**Table 1**). These data suggest that DR, which had the largest lower 95% CI bounds for the slope and for R_critDSB_, and one of the largest lower 95% CI bounds for the intercept (**Table 1**), has powerful mechanisms for resisting CIR as individual cells even when they are plated at low concentration, and also has a strong capacity for cooperative CIR protection. In other words, *intra*cellular and *extra*cellular antioxidant capacities, as well as DSB repair, appear strongly developed in DR.

The situation was less clear-cut with EC strains. For example, in EC1 an increase in growth-inhibitory critical dose rate was observed between -3 and -2 dilutions (**Fig 3**), which suggests that very small numbers of plated cells (approximately 1,000 to 10,000) were sufficient to “help” each other to cope with CIR. This resulted in a large lower 95% CI slope bound for EC1 (**Table 1**). In contrast, an increase in critical dose rate in EC2 was observed at much higher cell numbers than in EC1: between -1 and 0 dilutions, or approximately 10^5^ to 10^6^ cells. Since the number of cells required to “help” each other under CIR was much higher for EC2 than for EC1, the lower 95% CI slope bound for EC2 was correspondingly smaller than that for EC1 (**Table 1**) despite the fact that EC2 was much more CIR resistant than EC1 and tolerated higher dose rates (**Figs 1 and 3**).

Importantly, however, when both the lower and upper bounds of the slope CIs are considered, they overlap for EC1 and EC2 (**Table 1**). Therefore, there is insufficient evidence to rank EC1 and EC2 by *extra*cellular antioxidant capacities. The intercept for IR-resistant selectant EC2, however, was much larger than the one for wild-type EC1 (**Table 1**). This suggests that EC2 cells at low concentrations are similarly resistant to CIR as DR (with similar intercepts), and much more resistant than EC1 (**Table 1**). A plausible model-based conclusion is that EC2 cells have higher *intra*cellular antioxidant capacity and DSB repair capacity than EC1 cells, but the *extra*cellular antioxidant capacities of both strains may be similar (**Table 1**).

Among the three fungal species for which model-based analysis was informative, TM had the largest lower 95% CI bounds for the intercept and for R_critDSB_ (**Table 1**). These results suggest high intracellular antioxidant capacity and DSB repair capacity in TM. Such hallmarks of IR resistance are a new finding for this fungus. Slope CI bounds for the three fungi listed in **Table 1** were positive and overlapped each other. Consequently, there is evidence of cooperative CIR protection in these organisms, although a clear ranking among them was not possible due to large uncertainties in the slope estimates.

The growth-inhibitory dose rate for the remaining 4 microorganisms (EC3, CP, KE, PK) did not change with cell concentration (**Fig 3**). In these cases: (1) slope values were consistent with zero, indicating possible lack of cooperative radioprotection; (2) our model-based analysis could not reliably distinguish whether the intercept term (ROS-mediated effects) or the R_critDSB_ term (DSB-mediated effects) determined the growth-inhibitory critical dose rate. Model parameter estimates for EC3, CP, KE, and PK based on currently available data were therefore uninformative and are not discussed.

### Extracellular antioxidant capacities by oxygen radical absorbance capacity (ORAC) assay

**Fig 4:**
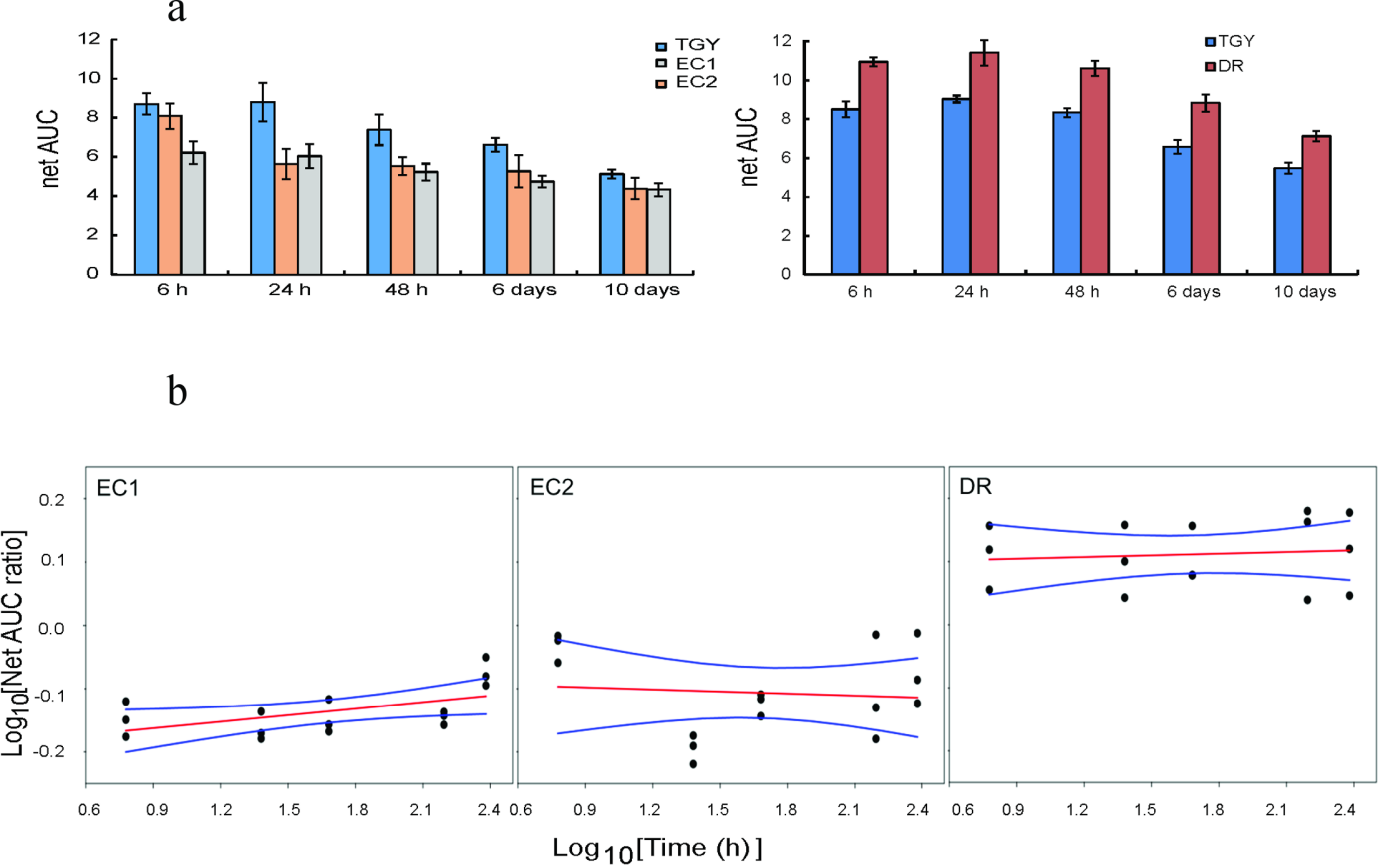
ORAC of TGY harvested with or without bacterial growth. **a**: The net AUC (net area under the fluorescence decay curve) is an integrative value of total fluorescence during antioxidant reaction in the presence of the indicated sample. **b:** Linear regression for log-transformed ratios of net AUC for samples with indicated bacteria to samples without bacteria, vs. log-transformed time. Red lines = regression best fits, blue lines = 95% confidence intervals. Y-axis values >0 suggest that the indicated microorganisms increased the ORAC of the medium; values <0 suggest the opposite—microorganisms decreased the ORAC.

DR, which was more CIR-resistant than EC1 and EC2 (**Figs 1 and 3**), also exceeded both EC strains in ORAC of their spent media (**Fig 4A**). These visually apparent differences between DR and EC strains were quantified by linear regression using log-transformed time (independent variable) and log-transformed net area under the ORAC fluorescence decay curve (AUC) ratio (dependent variable). A log-transformed net AUC ratio >0 indicates that bacteria increased the medium’s ORAC, whereas a ratio <0 indicates the opposite. The best-fit regressions and their 95% uncertainty bounds were clearly >0 for DR and <0 for EC1 and EC2 (**Fig 4B**). These results suggest that highly CIR-resistant DR likely scavenged more ROS in medium than the more CIR-sensitive EC strains. However, it is important to note that ORAC probably does not measure all relevant ROS types.

### Mixed culture experiments

**Fig 5:**
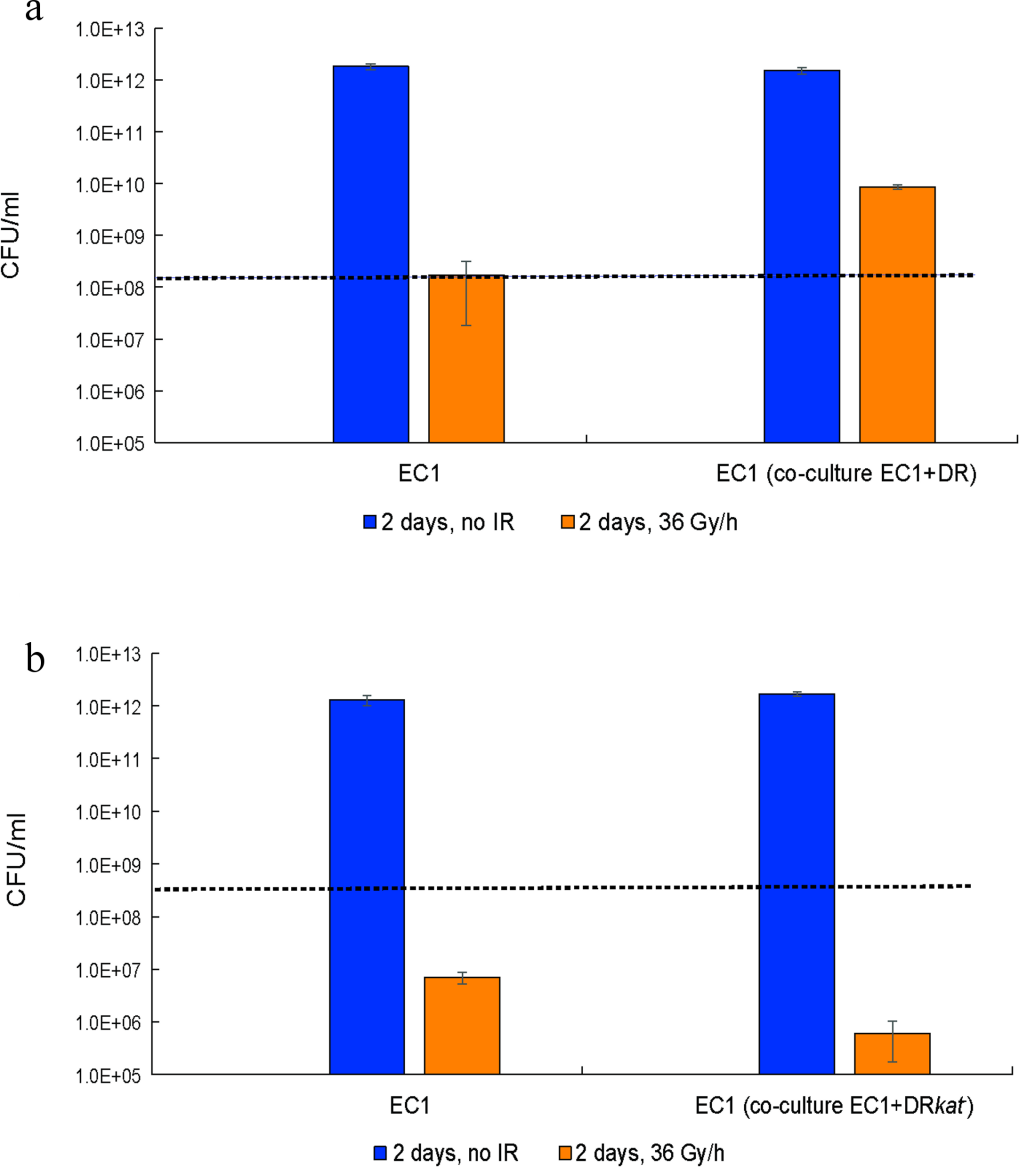
Microbial cooperation under CIR. **a**: Growth of EC1 in the presence or absence of 36 Gy/h for 2 days, either in pure culture or mixed in 1:1 co-culture with DR. **b**: As for panel A, but with DR*kat*^-^ substituting for DR. The y-axis shows clonogenically viable cell concentrations normalized to 1 ml: the actual numbers of viable cells are 200 times smaller because only 5 μl of each species were used in these experiments. Dashed lines indicate cell concentrations under the assumption of no net proliferation.

To determine if cell-cell interactions can modulate CIR resistance not only within species but also between different species from different phyla, we performed mixed culture experiments. Two-day exposure to 36 Gy/h CIR reduced the clonogenically-viable wild-type EC1 cell numbers by a factor of 1.90×10^−5^ (95% confidence intervals, CI: 1.73×10^−5^, 2.07×10^−5^), relative to unirradiated controls. In other words, unirradiated controls proliferated actively over 2 days, whereas proliferation of CIR-exposed EC1 cultures was essentially inhibited (**Fig 5A**). However, when EC1 and DR were co-cultured under CIR, EC1 proliferation was affected by a factor of only 4.90×10^−3^ (95% CI: 4.29×10^−3^, 5.53×10^−3^). Therefore, co-culture with wild-type DR strongly increased CIR-exposed EC1 clonogenicity: by a factor of 4.29×10^−3^/1.73×10^−5^ = 258 (95% CI: 226, 292) (**Fig 5A**). However, co-culture with DR*kat*- had the opposite effect: it reduced EC1 numbers by a factor of 1.9×10^−2^ (95% CI: 1.6×10^−2^, 2.2×10^−2^) (**Fig 5B**). By comparison, wild-type DR or DR*kat*^-^ had no appreciable effect on EC1 numbers when these organisms were co-cultured without CIR (**Fig 5B**).

### Comparison of sensitivities to CIR and AIR

There are few examples of either discordance or accordance between AIR and CIR responses because CIR studies on multiple species under the same controlled conditions are rare. There are, however, reports where resistance levels to AIR and CIR are not aligned [22,23]. For example, some microorganisms (*e*.*g*. DR) are extremely resistant to both AIR and CIR, whereas others are resistant to CIR but not AIR (*e*.*g*. *Lactobacillus plantarum*) [5,22,24,25]. In our study, the growth-inhibitory critical CIR dose rates for EC2 and for DR differ by only ~1.5-fold (**Figs 1 and 3**), whereas AIR doses which reduce survival to 10% (D_10_) for these same organisms differ by >50-fold (**S2 Fig**). Moreover, we show that DR*kat*^*-*^, which is as resistant to AIR as the wild-type [19], is much more CIR-sensitive than the wild-type (**Fig 2**). Below, we report that AIR- and CIR-resistance phenotypes are distinct among fungi as well as among prokaryotes.

Our detailed quantitative analysis of 145 fungi (**Table A in S1 File**) under the same conditions supports the conclusion that there is only a weak relationship between sensitivity to AIR and CIR, as follows. AIR D_10_ ranged from 0.1 to 6.5 kGy among the studied fungi (median = 1.0 kGy, 25^th^ percentile = 0.50 kGy, 75^th^ percentile = 2.0 kGy). As expected, those fungi with higher D_10_ were somewhat more likely to be CIR-resistant: 38.8% of fungi that fell into the first (lowest) D_10_ quartile could grow at 36 Gy/h, and for the second, third and fourth quartiles, the corresponding percentages were 48.4%, 66.7%, and 69.0%, respectively. The Pearson correlation coefficient between having D_10_ above the median (i.e. D_10_>1 kGy scored as 1, vs. D_10_≤1 kGy scored as 0) and the ability to grow under 36 Gy/h (scored as 1 for growth and 0 for no growth) was significant (p-value 2.3×10^−3^), but weak (Pearson correlation coefficient value only 0.251). These results suggest a trend for increasing CIR resistance with increasing AIR resistance, but that this trend was weak.

**Fig 6:**
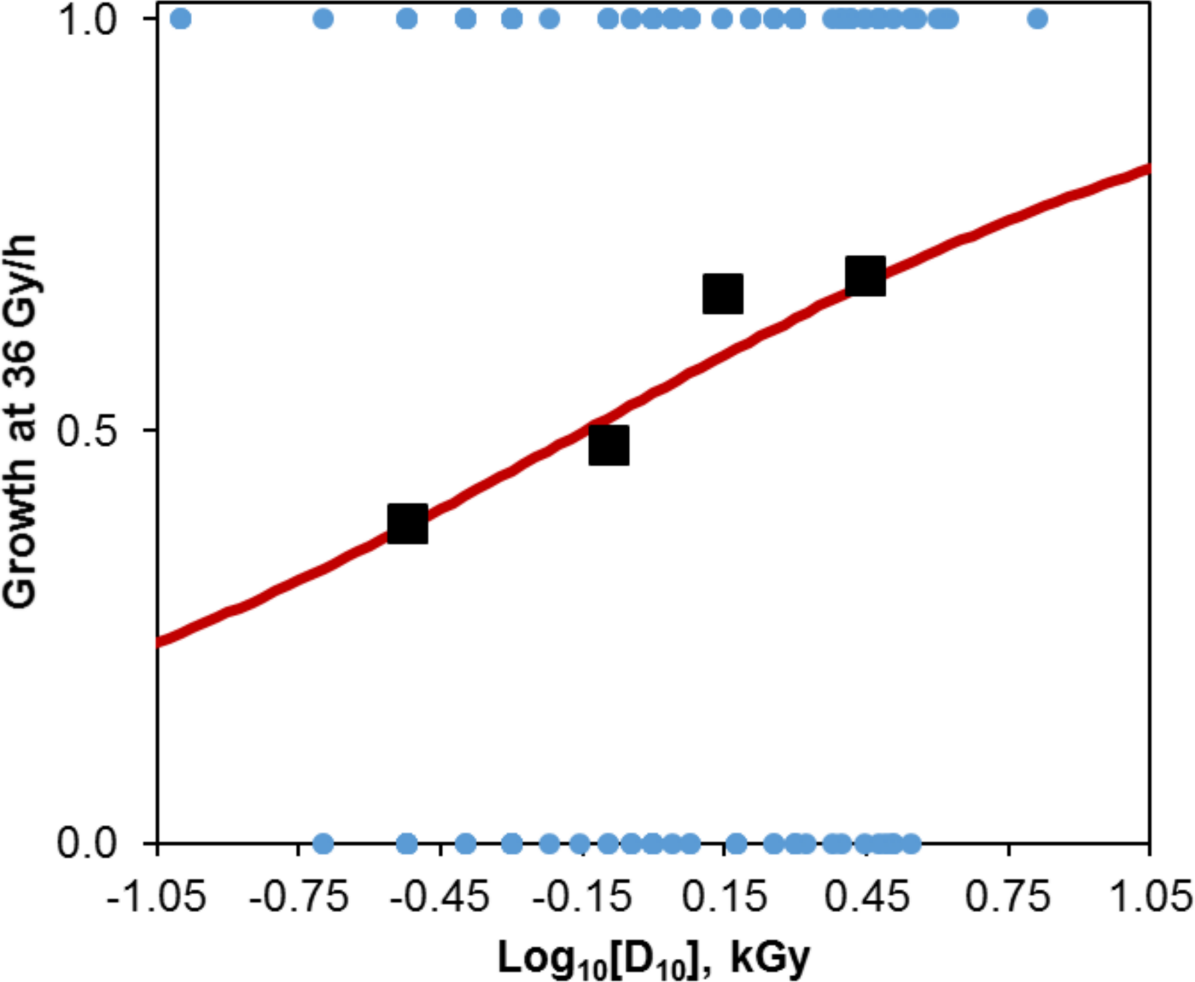
Quantification of responses to AIR and CIR for fungi. Logistic regression intended to predict growth at 36 Gy/h based on log_10_[D_10_]. D_10_ = AIR dose which kills 90% of population. Growth at 36 Gy/h was a binary variable (0 = no growth, 1 = growth). Blue circles indicate raw data; Black squares indicate summary data for log_10_[D_10_] quartiles, where x-axis shows median log_10_[D_10_] values for each quartile and y-axis shows fractions of fungi which grew under 36 Gy/h; Red curve = best-fit model predictions.

To assess the strength of this association between AIR and CIR sensitivity levels in greater detail, we performed logistic regression using log_10_[D_10_] as the independent variable and ability to grow under 36 Gy/h as a binary dependent variable (0 = no growth, 1 = growth). The best-fit parameter values were: “slope” = 1.26 (SE: 0.46, 95% CI from nonparametric bootstrapping: 0.30 to 2.29, p-value: 0.0067), “intercept” = 0.18 (SE: 0.17, 95% CI: -0.15 to 0.53, p-value: 0.28). Notably, the meanings of these parameters are different from the slope and intercept used in the mechanistic model of CIR effects (Eqs 8A and 8B). Here the “intercept” determines the probability to grow under 36 Gy/h for fungi with log_10_[D_10_] = 0, and the “slope” determines the dependence of this probability on log_10_[D_10_]. The best-fit “slope” value and its uncertainties indicate a significant positive association between log_10_[D_10_] and ability to grow under 36 Gy/h (shown graphically in **Fig 6**). However, the regression model had very low predictive power: McFadden’s pseudo R^2^ was only 0.039, which is much less than what is seen in models with strong predictive power–for such models this metric should generally be ≥0.20.

The area under the receiver operating characteristic (ROC) curve is a commonly used approach to quantify the performance of a binary classification system. In this case, the classification refers to whether or not a specific fungal strain was able to grow under 36 Gy/h. The area under the ROC curve for a completely random classification is 0.5. For our logistic regression model it was 0.65 –well below the range of strong performance (≥0.8). Cross-validation (3-fold) also showed a fairly high classification error rate of 37%, which suggests that the model often misclassified fungi as CIR-resistant when they in fact were not, or vice versa.

Members of the genus *Saccharomyces* composed a large fraction of the analyzed data set (49 out of 145 fungal strains), and therefore we repeated the analysis excluding this genus to ascertain how this changes the results. There was no qualitative difference: the regression “slope” estimate was 1.55 (SE: 0.60, 95% CI: 0.29, 3.05, p-value: 0.010) and McFadden’s pseudo R^2^ was 0.056.

More complicated models which included Phylum as a taxonomic variable (**Table A in S1 File**), or Phylum and Phylum×log_10_[D_10_] as predictors, in addition to log_10_[D_10_], had lower information theoretic support (higher Akaike information criterion scores) than the model with log_10_[D_10_] only, and none of these additional predictors achieved statistical significance.

Taken together, these quantitative assessments support the conclusion that there is only a weak relationship between AIR resistance and CIR resistance. The absence of a strong relationship implies that extrapolation from acute to chronic radiation effects may frequently lead to erroneous conclusions, probably because the sets of physiological adaptations need to resist AIR and CIR do not completely overlap.

## Discussion

Focus on DR as a model CIR-resistant organism was driven, in part, by the discovery of this bacterium beneath a million-gallon radioactive waste tank (SX-108) that has been leaking for over 50 years [1] and by its ability to grow under high-level CIR (60 Gy/h) in the laboratory [26]. Today, the most developed microbial treatment proposed for high-level radioactive sites involves DR. Our current findings represent an unexpected boost to the prospects of bioremediation of radioactive sites: (1) Extending earlier records set for growth under CIR, we show that some bacteria (DR, EC2) and fungi (TM) can grow under 126, 94 and 67 Gy/hour, respectively. (2) A mutant EC strain previously selected for AIR resistance (EC2) is extremely CIR-resistant (**Figs 1 and 3**). (3) Cooperative radioprotection between the model bacteria DR and EC under CIR is clear (**Fig 5**). Thus, multiple bioremediation-competent microorganisms, engineered or not, and held in microbial collections (*e*.*g*., ATCC, EX) could either be evolved for CIR resistance, or be introduced with wild-type DR in binary cooperation scenarios.

**Fig 7:**
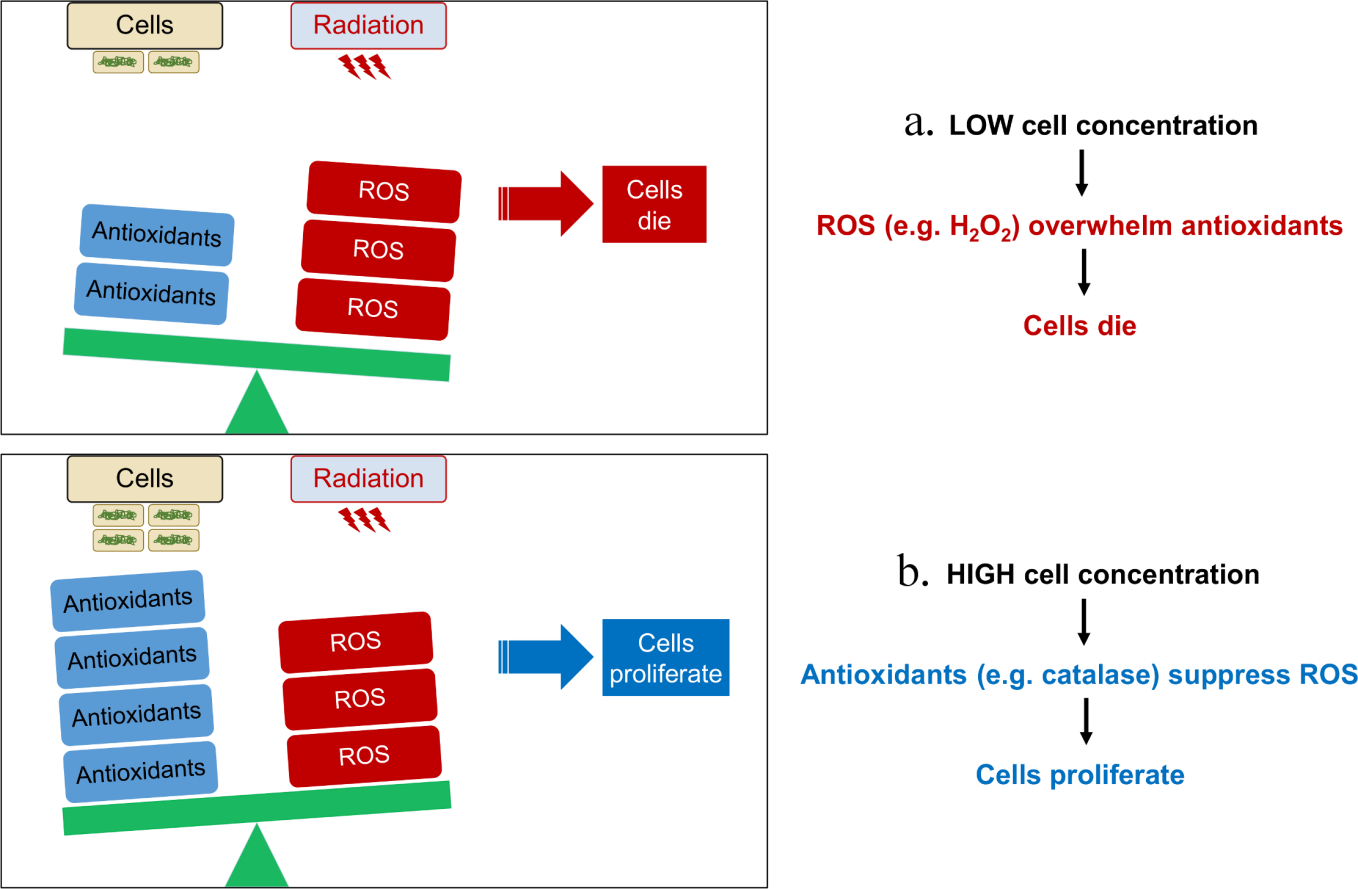
A schematic representation of the effects of cell concentration on microbial resistance to CIR.

Our mathematical modeling of bacterial and fungal growth under CIR predicts that a growth-inhibitory critical dose rate occurs when the rate of ROS detoxification is overwhelmed by the rate of IR-induced ROS production [20] (**Fig 7**). The critical dose rate is affected by many factors: the organism’s intracellular and extracellular antioxidant capacities, cell concentration, composition of growth medium, temperature, O_2_ concentration, etc. According to our model, the increase in growth-inhibitory critical dose rate with increasing cell concentration provides evidence for intercellular cooperation, as observed most notably for DR, EC2, and TM among those microorganisms where CIR resistance at different cell concentrations was studied in detail (**Fig 3**). This cooperation most likely involves collective radioprotection by cells through secretion of antioxidants into the medium.

Multiple generic and organism-specific mechanisms/antioxidants may contribute to intra- and inter-specific cooperative cell radioprotection and resistance to CIR [27]. First, our results (**Figs 2 and 5**) suggest an important role for the extremely efficient ROS-scavenging enzyme catalase, mediating H_2_O_2_ detoxification. These results are consistent with previous reports that DR, which exhibited strong cooperative cell radioprotection in our study, has very high catalase activity compared with other tested bacterial strains [20,28–33]. Dioxygen (O_2_) generation by catalase could also assist aerobic metabolism [34]. We showed that catalase is important for survival under CIR and for cross-species protection under CIR by our experiments involving the catalase negative DR mutant. This mutant was much more sensitive to CIR than the wild type but its resistance could be restored by exogenous catalase (**Fig 2**). The catalase negative DR mutant also failed to protect EC from CIR, whereas the wild type did protect EC (**Fig 5**).

Second, several tested microorganisms including DR secrete proteases which can diffuse through agar (**S3 Fig**). For example, our analysis of spent liquid media showed that DR secretes different proteases. In growth medium, proteases yield peptides, which become growth substrates and give rise to Mn antioxidants in DR [5,35].

Third, we measured antioxidants in the growth medium using the ORAC assay (**Fig 4**), which showed that in liquid TGY DR produced more antioxidants than either of the two tested EC strains. This difference between DR and EC in antioxidant production was clearly visible (**Fig 4A**) and supported by linear regression analysis (**Fig 4B**). Fourth, antioxidant enzymes can be induced in a cell-concentration-dependent manner through quorum sensing [28,29].

Our data demonstrate that the effects of CIR and AIR are distinct (**Table 2, Fig 6, Table A in S1 File**). We argue that the weakness of the association between AIR and CIR resistance (**Fig 6**) is caused by qualitative differences between acute and chronic radiation stresses: *amounts* of damage are important for AIR, whereas *rates* of damage production and repair are important for CIR. For example, one strategy to survive AIR may involve lengthy shutdown of growth/proliferation to allow slow accurate repair of macromolecules [23,30]. This strategy can fail against CIR because surviving continuous exposure requires simultaneous growth/proliferation and damage repair (**Table 2**). We conclude that AIR resistance (high D_10_) is not a strong predictor of CIR resistance (growth over 36 Gy/h) in fungi (**Fig 6**). Many factors other than D_10_ and phylogenetics are likely to be involved in CIR resistance, which is *not* predicted by a genome sequence [36].

**Table 2:** Summary of the main findings of this study and of their interpretations.

**Finding 1: The cells of some microorganisms (from the same species or even from different species) can cooperate in resisting CIR.**
**Supporting evidence:** The critical CIR dose rate for several tested organisms (e.g. DR) increased markedly with increasing number of cells plated on a solid medium (**Figs 1 and 3**).
In mixed co-culture, wild-type DR (but not a catalase-negative DR*kat*^-^ mutant), strongly (by >200-fold) enhanced the survival of EC1 under CIR (**Fig 5**).
Exogenous catalase enhanced the survival of DR*kat*- under CIR (**Fig 2**).
**Interpretations:** Chemical detoxification of the growth medium by cells can explain these effects. For example, IR-resistant cells can detoxify IR-induced ROS in their surroundings, thereby helping nearby cells, including those from more IR-sensitive species. A mathematical model based on ROS and antioxidant production and removal kinetics provided an adequate quantitative description of the data for all tested microorganisms. In the model, an organism’s intracellular antioxidant capacity affects the critical CIR dose rate at low cell concentrations, whereas the extracellular capacity determines how the critical dose rate increases with increasing cell concentration.
**Finding 2: Extrapolating from AIR to CIR, or vice versa, can be unreliable.**
**Supporting evidence:** Resistance to AIR (D_10_) was not a strong predictor of the ability to grow under high-level CIR in fungi (**Fig 6**) and bacteria.
Co-culture with wild-type DR increased post-AIR survival of EC1 only marginally (**S4 Fig**), whereas DR strongly protected EC1 from CIR (**Fig 5**).
**Interpretations:** Acute and chronic IR stresses are qualitatively distinct, and resistance to one does not necessarily imply resistance to both. For example, one strategy to survive AIR may involve lengthy shutdown of growth/proliferation to facilitate repair of macromolecules. This strategy can fail against CIR because surviving continuous exposure requires simultaneous growth/proliferation and damage repair.
**Finding 3: Many fungi are highly resistant to AIR and CIR.**
**Supporting evidence:** Among 145 phylogenetically diverse fungal strains tested under the same conditions, the median D_10_ was 1.0 kGy, and >53% of these strains could grow under 36 Gy/h (**Table A in S1 File**).
**Interpretation:** DSB induction by IR is approximately proportional to genome size. Fungi generally have larger genomes than bacteria (**Table B in S1 File**), and maintaining a high efficiency of their DSB repair proteins may be more important for organisms with larger genomes in general. Radioresistant fungi, many of which probably remain undiscovered, may have useful properties for radioactive waste bioremediation and in harnessing their antioxidants for radioprotective purposes.

Because the DSB yield per IR dose per Mbp of DNA is similar across phylogenetic groups [34,37], organisms with larger genomes (*e*.*g*. fungi, 12–20 Mbp per haploid genome in this study) suffer more DSBs per unit time at the same CIR dose rate than organisms with smaller genomes (*e*.*g*. bacteria, 3–5 Mbp) [38]. Our finding that the growth-inhibitory critical dose rates for IR-resistant fungi and bacteria (*e*.*g*. TM and DR) differ by a smaller factor (**Figs 1 and 3**) than their genome sizes, therefore suggests that some fungi can deal with larger numbers of CIR-induced DSBs (**Table B in S1 File**). A similar pattern occurs when comparing AIR-induced DSB yields at D_10_ doses. These results are consistent with reports of extremely IR-resistant fungi, including representatives isolated from high-level IR sites in Chernobyl [39–42]. A likely explanation is that the fractional contribution of DSBs to IR-induced mortality is in general larger for cells with larger genomes. Therefore, cells with larger genomes evolved more efficient DSB repair systems (**Table 2**) [43], where DSB repair efficiency depends on the antioxidant status of cells [38].

Our comparative analysis of experimental and theoretical results provides fresh insight into the effects of CIR on prokaryotic and eukaryotic cells. The bacterium DR was highly protective of its neighbors under high-level CIR, even when those neighbors were IR-sensitive species of a different phylum (EC). The dramatic >200-fold CIR-protection of EC1 by DR probably occurred because antioxidants secreted by DR detoxified radiogenic ROS (*e*.*g*. H_2_O_2_) in the growth medium. DR*kat*^-^, however, inhibited EC1 growth under CIR (**Fig 5B**), probably because DR*kat*^-^, like other Mn-accumulating catalase-negative organisms (*e*.*g*. *L*. *plantarum*), releases H_2_O_2_ [34]. Such “toxicity” of DR*kat*^-^ towards EC1 can be considered a form of “radiation bystander effect”. As reported previously [22], catalase does not play a significant role in AIR survival. Consistently, we showed that EC1 killing by AIR is only marginally reduced when EC1 was mixed with DR (**S4 Fig**).

When assessing the effect of IR hazards, our results clearly demonstrate the rationale for studying CIR responses instead of AIR responses in cells exposed to environmentally-relevant dose rates that can match or exceed those used in this study (*e*.*g*. close to nuclear plant accident sites such as Fukushima). Any number of naturally CIR-sensitive environmental bacteria suitable for bioremediation of DOE sites could be tested together with CIR-resistant bacteria (*e*.*g*. DR) or fungi (*e*.*g*. TM) in binary bioremediation scenarios under high-level CIR. In such situations, CIR-resistant microorganisms could allow other species that are more CIR-sensitive but able to detoxify radioactive wastes. Importantly, CIR-resistant bacteria and fungi could cooperatively protect the human gastrointestinal tract from CIR-induced toxicity and/or carcinogenesis.

## Materials and methods

### Bacterial and fungal strains

For determination of growth-inhibitory critical CIR dose rates at different cell concentrations we used the following bacteria (**Table B in S1 File**): wild-type *Escherichia coli* (EC1); *E*. *coli* strains (EC2 and EC3) selected for AIR resistance by directed evolution of EC1 [14]; wild-type DR and a catalase-A-defective mutant DR*kat*^*-*^ (deficient in DR1998) [19]. In addition, we used six wild-type fungal species: *Candida parapsilosis* (CP), *Kazachstania exigua* (KE), *Pichia kudriavzevii* (PK), *Rhodotorula lysinophila* (RL), *Saccharomyces cerevisiae* (SC), and *Trichosporon mucoides* (TM) (**Table B in S1 File**).

To investigate quantitatively the relationship between resistance to AIR and CIR, we measured the AIR dose that clonogenically kills 90% of the cells (D_10_) and the ability to grow under 36 Gy/h CIR in 145 phylogenetically diverse fungi on YPD medium (pH 7.0) (**Table A in S1 File**).

### Culture conditions

Bacterial cells were grown in liquid TGY medium (1% bactotryptone, 0.5% yeast extract, and 0.1% glucose) to OD_600_ ~0.9 (~2 × 10^8^ Colony Forming Units (CFU)/ml) at 32 ^o^C. For solid TGY medium, 1.5% w/v bactoagar was added. Fungal strains were pre-grown in liquid YPD (2% bactopeptone, 1% yeast extract, and 2% glucose) at 26 ^o^C. OD_600_ was adjusted to ~0.8 (~1 × 10^8^ CFU/ml). For solid YPD medium, 2% w/v bacto agar was added.

### Irradiation with γ-rays

Bacteria and fungi were exposed to CIR (0–180 and 0–126 Gy/h, respectively) under aerobic or microaerobic (bacteria only) growth conditions at ~26°C within ^137^Cs irradiators (JL Shepherd and Associates, Mark I Model 68 A, S.N. 1064, San Fernando, CA, USA; and Gammacell 40 irradiation unit, Atomic Energy of Canada Limited, Ottawa, ON, Canada). Microaerobic conditions, as operationally defined for the purposes here, were achieved by sealing Petri dishes with Parafilm “M” laboratory film.

For each organism and CIR dose rate, 5 μl of cell suspension were aliquoted (spotted) onto the surface of solid medium, followed by CIR exposure. As mentioned previously, six different sequential log_10_ dilutions of the cell suspensions, called 0 to -5 dilutions, respectively, were used. They contained approximately 10^6^, 10^5^, 10^4^, 10^3^, 10^2^, and 10^1^ cells.

For AIR exposures, cells were irradiated with ^60^Co at ~12 kGy/h (JL Shepherd and Associates model 109–68 irradiator, San Fernando, CA, USA) aerobically on wet ice in liquid media at ~10^8^ CFU/ml. Such conditions minimize the impact of metabolically-induced ROS and DNA repair during exposure [30]. Irradiation was followed by CFU counts.

### Quantification of growth under CIR

Visual inspection and analysis of cell growth by ImageJ software (https://imagej.nih.gov/ij/docs/intro.html, S1 Methods) allowed us to estimate the lower and upper limits of the growth-inhibitory critical CIR dose rate for studied bacterial and fungal strains. For example, suppose that an undiluted inoculum of EC1 produced some growth (individually identifiable colonies or a lawn) at 36 Gy/h, but no growth at 67 Gy/h. Then the critical dose rate for this organism at this cell concentration was between 36 Gy/h and 67 Gy/h.

To assess the strength of the association between AIR and CIR sensitivity levels on the larger data set of 145 phylogenetically diverse fungi, we performed logistic regression (by R 3.2.3 software, [44]) using log_10_[D_10_] as the independent variable and ability to grow under 36 Gy/h as a binary dependent variable (0 = no growth, 1 = growth).

### Extracellular ROS absorbance capacities by ORAC

We used the oxygen radical absorbance capacity (ORAC) assay [45] to measure small-molecule antioxidants in spent TGY medium. The net area under the fluorescence decay curve (AUC) was measured at several time points (6 hours to 10 days after the start of the experiment) using the <3 kDa fraction of the TGY medium alone (control) and for media where indicated microorganisms (EC1, EC2 or DR) were grown. Each organism was grown at its optimal temperature.

### Mixed culture experiments

EC1 was irradiated at 36 Gy/h: alone (in pure culture), or mixed in 1:1 proportion with wild-type DR or with catalase-A-negative DR*kat*^-^ [19]. EC1 and DR were pre-grown up to OD_600_ ~0.9, from these cultures we prepared: (1) 500 μl TGY + 500 μl DR culture; (2) 500 μl TGY + 500 μl EC1 culture; and (3) 500 μl DR culture + 500 μl EC1 culture. Five μl of culture-containing liquid was spotted onto separate TGY plates. After 2 days of CIR, the colonies or bacterial lawns were harvested and serial dilution was performed in TGY medium to estimate the concentrations of clonogenically viable EC1 (pigmentless) and DR (red) cells. We statistically analyzed these data (using Maple 2016® software) by assuming that the colony counts were Poisson-distributed random variables (S1 Methods, Equations C-E).

### Catalase administration under CIR

We compared wild-type DR and DR*kat*^*-*^ growth under CIR. Both bacteria were grown up to OD_600_ ~0.9, followed by dilution and 5 μl culture were spotted onto TGY medium. We also evaluated if purified catalase can improve the survival of DR*kat*^-^ under CIR: 5 μl catalase (20 mg prot./ml) were dispensed onto the middle of TGY plates containing DR*kat*^-^ cells immediately before irradiation began.

### Mechanistic mathematical model

The main assumptions of our mathematical model were described in previous publications [20,21,46]. The model is not intended to describe in a detailed way the multiple types of oxidants and antioxidants relevant for cell survival, as has been done in previous studies [13,47]. Instead, our goal is to capture the main general features of ROS/antioxidant interactions under CIR by using the following simplified and manageable approach.

Briefly, we assume that cell proliferation during CIR is possible only if the rate of ROS detoxification by intracellular and extracellular antioxidants is faster than the rate of radiogenic ROS production (the effects of DNA damage from radiation energy deposition on or very close to DNA are analyzed separately, as discussed below). If the rate of ROS production exceeds the rate of detoxification, a threshold is reached: above it, ROS steadily accumulate and kill the cells. The threshold occurs at a critical CIR dose rate.

We assume that ROS are detoxified by enzymatic and non-enzymatic antioxidants [5,43], generically called *A* here, and by reacting with molecules not critical for cell survival. These assumptions are represented by the following system of differential Eqs (1A–1C) which track the rates of change of oxidant/antioxidant concentrations within/in the vicinity of the average cell:

$$\frac{dROS(t)}{dt}=c_{1}\;R–c_{2}\;ROS(t)A(t)–c_{3}\;ROS(t),$$ (1A)

$$\frac{dA(t)}{dt}=–c_{2}\;ROS(t)A(t)+c_{4}\;ROSC(t),$$ (1B)

$$\frac{dROSC(t)}{dt}=c_{2}\;ROS(t)A(t)–c_{4}\;ROSC(t)$$ (1C)

Here *A* = active form of the antioxidant, *ROSC* = ROS-antioxidant interactant (temporarily inactive form of the antioxidant), *R* = CIR dose rate, *c*_*1*_ = ROS production by CIR, *c*_*2*_ = ROS removal by antioxidant, *c*_*3*_ = ROS removal by reactions with non-critical molecules, *c*_*4*_ = regeneration of active antioxidant.

To simplify Eqs (1A–1C), we made the following assumptions: (1) The turnover between active and inactive forms of the antioxidant (*A* and *ROSC*, respectively) is fast. Therefore, both forms of the antioxidant exist in equilibrium, and the sum of their concentrations is equal to the total antioxidant concentration (*A*_*tot*_). (2) *A*_*tot*_ is constant (independent of dose/dose rate) because under severe IR exposure antioxidants are maximally induced.

Implementing these assumptions allows us to set d*A*(t)/dt = d*ROSC*(t)/dt = 0 and to solve Eqs 1B and 1C) for the equilibrium concentrations of *A* and *ROSC*. Substituting the solutions into Eq (1A) generates the following equation for ROS:

$$\frac{dROS(t)}{dt}=c_{1}\;R–c_{2}c_{4}A_{tot}\frac{ROS(t)}{\left[c_{4}+c_{2}\;ROS(t)\right]}–c_{3}\;ROS(t)$$ (2)

Next, we assume that ROS production/removal kinetics are much faster than cell proliferation. Therefore, ROS exist at an equilibrium concentration ROS_eq_. This allows us to set d*ROS*(t)/dt = 0 and to solve Eq (2). The solution for ROS_eq_ is given by the following expressions:

$${ROS}_{eq}=\frac{c_{2}X_{1}–c_{3}c_{4}+\sqrt{X_{2}}}{2\;c_{2}c_{3}},$$ (3A)

$$X_{1}=c_{1}\;R–c_{4}\;A_{tot},$$ (3B)

$$X_{2}={\left(c_{2}X_{1}\right)}^{2}+2c_{2}c_{3}c_{4}X_{3}+{\left(c_{3}c_{4}\right)}^{2},$$ (3C)

$$X_{3}=c_{1}\;R+c_{4}\;A_{tot}$$ (3D)

Importantly, we assume that the cell concentration and the intracellular and extracellular antioxidant capacities of the cells affect ROS/antioxidant kinetics. If cells secrete extracellular antioxidants, the concentration of such antioxidants in the growth medium should increase with increasing cell concentration. This should result in an increased ROS detoxification rate, thereby increasing the cell culture’s CIR resistance and allowing the cells to grow at a higher dose rate. This occurs under H_2_O_2_ exposure [13].

We model these processes using the following equation, where *N* is the initial cell concentration (just before irradiation begins), and a_1_ and a_2_ are the intracellular and extracellular antioxidant capacities, respectively:

$$A_{tot}=a_{1}+a_{2}N$$ (4)

This expression for A_tot_ can be substituted back into the equation for ROS_eq_ (Eqs 3A–3D). Viewed from the perspective of a given cell, the term *a*_1_ represents the antioxidant concentration within this cell when there are no other cells in the vicinity. When other cells are present and secrete/generate antioxidants in the extracellular medium, the term *a*_2_ represents their contribution to the antioxidant concentration within the selected cell (*e*.*g*. by diffusion of extracellular antioxidants into the cell).

Our main goal here is to predict the growth-inhibitory critical dose rate (R_crit_) at which antioxidants will be overwhelmed and ROS_eq_ will become too large to allow cellular proliferation. This is done by solving the equation dROS_eq_^3^/dR^3^ for R, which has the following solution:

$$R_{crit}=c_{4}([a_{1}+a_{2}N]c_{2}-c_{3})/(c_{1}c_{2})$$ (5)

In addition to ROS-mediated effects of IR, it is also important to consider DNA double strand break (DSB)-induction. A simple way of doing so is to assume that proliferation occurs only if the IR-induced DSB production rate remains below a critical threshold, DSB_crit_. The value of DSB_crit_ can depend on multiple factors, *e*.*g*. rates/fidelities of DSB repair processes, duration of DNA damage response-induced cell proliferation arrest [23,48]. Assuming that DSB induction is proportional to dose rate, we write the following equation where DSB_y_ is the DSB yield per cell per Gy and R_critDSB_ is the critical IR dose rate from DSB effects:

$$R_{critDSB}={{DSB}_{crit}/DSB}_{y}$$ (6)

The total growth-inhibitory critical dose rate R_critTot_ from both ROS-mediated and DSB-mediated IR effects is the minimum of the values based Eqs 5 and 6. It can be expressed as follows, where Minimum is the function that returns the smallest value of its arguments:

$$R_{critTot}=Minimum[{R_{critDSB},R}_{crit}]$$ (7)

Eq (7) specifies that cells can proliferate only if the rates of *both* ROS detoxification, and DSB rejoining, are not overwhelmed by CIR.

Under physiological conditions, the contribution of CIR-induced ROS (Eq 5) to cellular damage is greater than the contribution of direct actions of γ-photons [11,49–51]. Moreover, ROS-mediated effects can be modulated by cell-cell interactions, unlike direct effects. According to the model, this ROS-mediated contribution R_crit_ is a linear function of total antioxidant concentration, which in turn linearly depends on the cell concentration (Eq 4). The “intercept” is the value of R_crit_ when the cell concentration is close to zero: the predicted growth-inhibitory critical dose rate when only one cell is present and there are no neighboring cells to contribute antioxidants to the growth medium. It is calculated by substituting N = 0 into Eq (5). The “slope” is the rate of increase of R_crit_ with increasing cell concentration. It is calculated by differentiating Eq (5) over N. The solutions for the intercept and slope are below:

$$Intercept=c_{4}a_{1}/c_{1}-{c_{3}c}_{4}/(c_{1}c_{2})$$ (8A)

$$Slope=c_{4}a_{2}/c_{1}$$ (8B)

The assumptions underlying Eqs 8A and 8B eliminate the need to explicitly model the complicated kinetics of oxidant/antioxidant interactions and cell proliferation. Many adjustable parameters, which characterize such kinetics but are not the focus of this study, are avoided. Instead, Eqs 8A and 8B focus on the main parameters of interest: organism-specific differences in antioxidant capacities a_1_ and a_2_. The intercept depends on the intracellular antioxidant capacity a_1_, whereas the slope depends on the extracellular antioxidant capacity a_2_.

### Estimation and interpretation of model parameters

The model was fitted (as described in S1 Methods, Eqs A-B) to observed growth-inhibitory critical dose rate values (R_critOBS_) for each organism at each cell concentration. The model fits allowed us to estimate 3 important parameters for each organism: intercept and slope for ROS-mediated IR effects (Eqs 8A and 8B), and R_critDSB_ for DSB-mediated effects (Eq 6).

Importantly, if the data suggest an increase in growth-inhibitory critical dose rate with increasing cell concentration, the model generates a positive slope estimate: an indication that the studied organism secretes extracellular antioxidants. Therefore, according to the model, an organism’s intracellular antioxidant capacity affects the critical dose rate at low cell concentrations, whereas the extracellular capacity determines how the growth-inhibitory critical dose rate increases with increasing cell concentration.

In contrast, if the data show no change in growth-inhibitory critical dose rate with cell concentration, the role of extracellular antioxidants is predicted to be negligible. The model suggests that the limit on proliferation under CIR is the same at all cell concentrations because it is imposed either by intracellular antioxidant capacity or by the efficiency/speed of DSB repair.

## Supporting information

S1 Methods S1 Methods. (DOCX)

S1 Fig Microaerobic growth under CIR and recovery of microorganisms. **a**: Bacterial growth in microaerobic conditions under CIR. **b**: Post-CIR recovery of bacteria under microaerobic (parafilm-sealed plates) and aerobic (unsealed plates) conditions. **c**: Post-CIR recovery of fungi under aerobic conditions.
(TIF)

S2 Fig Clonogenic survival of bacterial strains exposed to AIR. Green: EC1; orange: EC2; red: EC3. Inset: DR.
(TIF)

S3 Fig Protease activity assessment in bacteria and fungi. **For bacteria the assay was performed on the beef agar, for fungi on YPD agar.** Halos indicate the presence of active proteases.
(TIF)

S4 Fig Survival of EC1 and DR exposed to AIR. Blue circles: EC1 survival in pure culture; blue triangles: EC1 survival in mixed EC1+DR culture; red circles: DR survival in pure culture; red triangles: DR survival in mixed EC1+DR culture.
(TIF)

S1 File Contains the following supplementary tables: Table A. Sensitivity to AIR and CIR, measured by D_10_ and the ability to grow at 36 Gy/h, respectively, in 145 phylogenetically diverse fungi. Table B. Estimates of DNA DSB repair capabilities of the tested organisms.
(DOCX)
