# Supplementary material for: Microbial cells can cooperate to resist high-level chronic ionizing radiation
Source: PLoS One. 2017 Dec 20;12(12):e0189261. doi: 10.1371/journal.pone.0189261 (PMC5738026; doi:10.1371/journal.pone.0189261)
Supplement: S1 Methods — (DOCX) [file pone.0189261.s001.docx]

**Protease secretion assays**

Secretory protease activities were tested on protease detection media plates [1]. Twenty-five ml of beef agar (0.3% beef extract (Sigma), 0.5% bacto peptone (BD), 1.5% bacto-agar (BD)) for bacteria and YPD with 1.5% bacto-agar for fungi were poured per square plate (12 × 12 cm) and allowed to solidify. 12 ml of top agar (3.3% skimmed milk powder (Bio-Rad), 0.25% beef extract, 0.4% bacto peptone, 1.25% bacto-agar for bacteria and YPD with 1.2% bacto-agar for fungi were poured after bottom layers solidified. Plates were inoculated by 1 µl loop of cultures from TGY plates for bacteria and YPD plates for fungi. Inoculated plates were incubated for 48-72 hours at 32 ^o^C for bacteria and 25 ^o^C for fungi. The protease activity was detected by visible halo (milk degradation).

**Quantification of growth under CIR**

We analyzed the photographs of bacterial and fungal growth under CIR by ImageJ software (National Institutes of Health, version 1.50e). Color photographs were converted to 8-bit grayscale, and the background was subtracted three times by using the “rolling ball” algorithm. A circular image area corresponding to the location on the plate where microbial cells have been seeded was selected. The darkness (mean and standard deviation) of the pixels within the selected area was quantified. Robust growth was considered to occur if the mean darkness minus 1.96 standard deviations was larger than zero, i.e. if the location where cells were originally seeded became much darker than the surrounding areas.

**Mechanistic mathematical model**

**Model fitting procedure**

As described in the main text, the experimental data allowed us to estimate the lower and upper limits of the observed growth-inhibitory critical dose rate R_critOBS_ for each organism at each of the six tested cell concentrations (i.e. log_10_-dilutions 0, -1, -2, -3, -4, and -5). For example, we found that R_critOBS_ for EC1 grown under aerobic conditions (on parafilm-unsealed plates) was between 36 and 67 Gy/h for dilutions 0, -1 and -2, and between 13 and 36 Gy/h for dilutions -3, -4 and -5. Fitting model predictions for the critical radiation dose rate (R_critTot_) to such observed data involved searching for values of the 3 parameters - intercept and slope from Equations (8A-B) and R_critDSB_ from Equation (6) – which generated a dependence for R_critTot_ that fit inside the intervals bounded by the lower and upper limits of R_critOBS_ at the tested cell concentrations. The dependence for predicted R_critTot_ was defined as follows, where dil is the log_10_ of the dilution factor:

$R_{critTot}=min(R_{critDSB},intercept+slope {10}^{dil})$ (A)

For example, an intercept of 22.5 Gy/h, a slope of 28.38×10^2^ Gy/(h×c*) and an R_critDSB_ of 52.4 Gy/h produced predictions which fit inside all six 6 R_critOBS_ intervals for EC1 listed above. However, this is not a unique solution and other parameter value combinations could also satisfy the condition of fitting within the R_critOBS_ intervals. For each organism, we searched for such solutions by Monte Carlo simulation using Maple 2016® software, as described below.

We fitted model predictions (Equation A) as follows. For each organism, values for log_10_ of the intercept and for log_10_ of R_critDSB_ were drawn at random from a uniform distribution bounded by 0 and 3. For the slope, log_10_ values were drawn at random from a uniform distribution bounded by 0 and 6. The lower and upper bounds for these uniform distributions were chosen to represent plausible limits for the corresponding variables. Then the intercept, slope and R_critDSB_ values were converted from the logarithmic to the linear scale so that they could be substituted into Equation A. The sign for slope values was randomly assigned to – or + with equal probability by a random number generator. Negative values for the slope are of course biologically meaningless, but they were generated to test whether the 95% confidence intervals (CIs) for the slope would overlap zero. For each random parameter combination, predicted R_critTot_ values were calculated at each dilution. If the predicted R_critTot_ value fell outside the R_critOBS_ intervals at a particular dilution, this was scored as a deviation from the data.

The R_critOBS_ value at the -5 dilution was considered less reliable than those at other dilutions because the number of plated cells at the -5 dilution was small (~10) and, therefore, there was a non-negligible probability of stochastic extinction of all these cells even at dose rates below the deterministic critical threshold. Consequently, deviations of predictions from the data were scored only at -4 to 0 dilutions. The number of deviations was summed over these dilutions, and the sum could therefore vary between 0 and 5 (corresponding to the results at 0, -1, -2, -3 and -4 dilutions). Only those intercept and slope combinations which produced the minimum sum of deviations (0 in most cases, or 1 if no solutions yielding 0 deviations were found) were retained. Monte Carlo simulations were continued until 1000 retained intercept and slope combinations accumulated for a given organism – usually 3,000,000 simulations were sufficient for this purpose. Summary statistics (standard deviation and 95% CIs) were calculated for the retained parameter values. For bacteria, the calculations were performed separately for parafilm-sealed and unselaled plates.

This approach produced multiple parameter combinations which were consistent with as many R_critOBS_ intervals as possible for the given organism. To find among them a single “best-fit” solution, we identified the parameter combination which had the smallest sum of squared differences (SS) between R_critTot_ predictions (from Equation A) and the lower and upper limits of R_critOBS_ intervals. The SS for a given organism is described by the following equation, where R_critOBSlow_ and R_critOBShigh_ are the observed lower and upper limits, respectively:

$SS=\sum_{dil=-4}^{0} {(R_{critTot}-R_{critOBSlow})}^{2}+({R_{critTot}-R_{critOBShigh})}^{2}$ (B)

**Mixed culture experiments**

We used the following approach to estimate the concentrations (C) of clonogenically-viable cells in mixed-culture experiments (wild-type DR + EC1 or catalase-negative DR*kat^-^* + EC1) with and without CIR exposure. According to the Poisson distribution with a mean value of µ, the probability *P(k)* of observing *k* colonies is given by the following equation:

$P\left( k \right)=\mu^{k}e^{-\mu}/k!$ (C)

For convenience, it is useful to work with the logarithm of this distribution to generate the log-likelihood (*LL*) function as follows:

$LL=\ln\left[ P\left( k \right) \right]=k ln\left[ \mu\right]-\mu-ln[k!]$ (D)

For our purposes here, the mean number of colonies µ(*i*) at the *i*-th serial dilution *D_ser_*(*i*) is predicted by the expression µ(*i*) = *C*×*D_ser_*(*i*), where *C* is the cell concentration which we would like to estimate. The term ln[*k*(i)!] has no value for estimating *C*, and therefore it can be omitted. To estimate the value of *C* we minimized the following sum of log-likelihoods, which was obtained by substituting µ(*i*) = *C*×*D_ser_*(*i*) into Equation D:

${LL}_{sum}=\sum_{i} k(i) ln\left[ CD_{ser}(i) \right]-CD_{ser}(i)$ (E)

This procedure was performed separately for EC1 and DR (wild type or *kat^-^*) for each experimental condition using the sequential quadratic programming (SQP) algorithm implemented in Maple 2016® software. Uncertainties (95% CIs) of the concentration estimates were calculated by profile likelihood. Uncertainties of concentration ratios (e.g. the ratio of EC1 cell concentrations with CIR vs without CIR) were estimated using Gaussian error propagation rules. This should be a reasonable approximation here because the colony counts on which the cell concentration assessment was based were typically large numbers, for which the Gaussian distribution closely approximates the Poisson distribution.

**References**

1. Ghosal D, Omelchenko M V, Gaidamakova EK, Matrosova VY, Vasilenko A, Venkateswaran A, et al. How radiation kills cells: survival of Deinococcus radiodurans and Shewanella oneidensis under oxidative stress. FEMS Microbiol Rev. 2005;29: 361–75. doi:10.1016/j.femsre.2004.12.007
