# Supplementary figures and images for: Microbial cells can cooperate to resist high-level chronic ionizing radiation
Source: PLoS One. 2017 Dec 20;12(12):e0189261. doi: 10.1371/journal.pone.0189261 (PMC5738026; doi:10.1371/journal.pone.0189261)

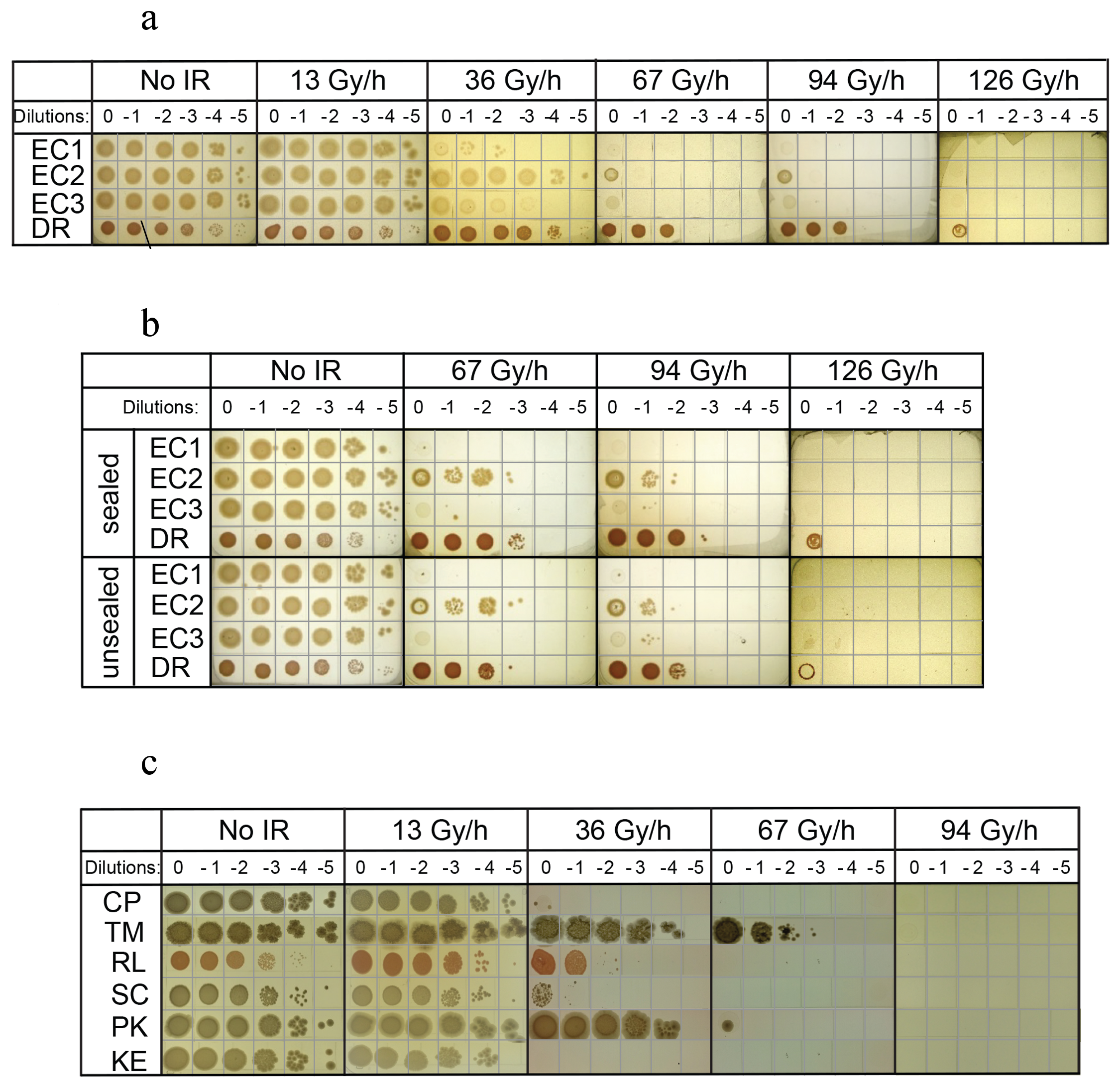

Supplement: S1 Fig — a: Bacterial growth in microaerobic conditions under CIR. b: Post-CIR recovery of bacteria under microaerobic (parafilm-sealed plates) and aerobic (unsealed plates) conditions. c: Post-CIR recovery of fungi under aerobic conditions. (TIF) [file pone.0189261.s002.tif]

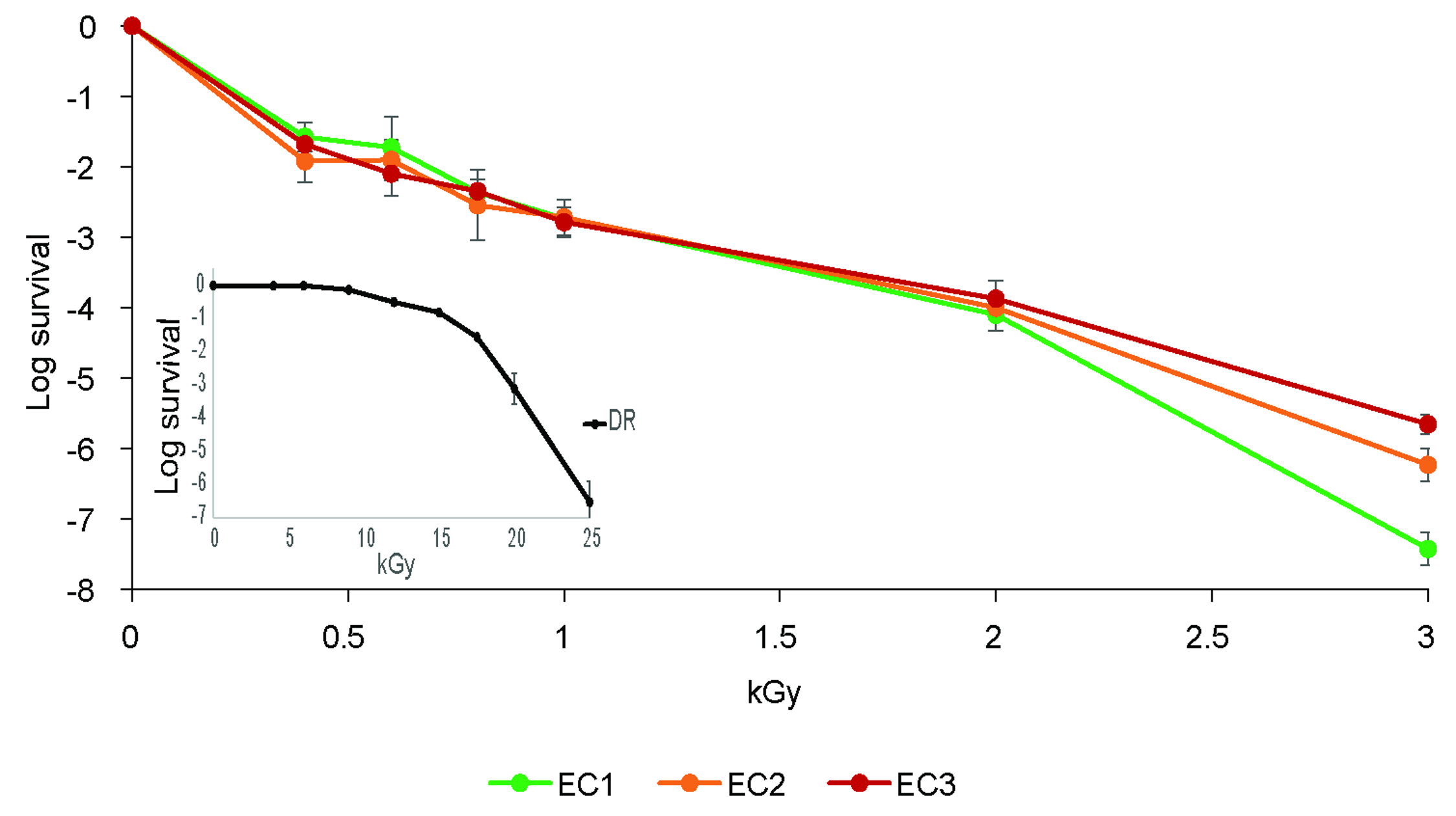

Supplement: S2 Fig — Green: EC1; orange: EC2; red: EC3. Inset: DR. (TIF) [file pone.0189261.s003.tif]

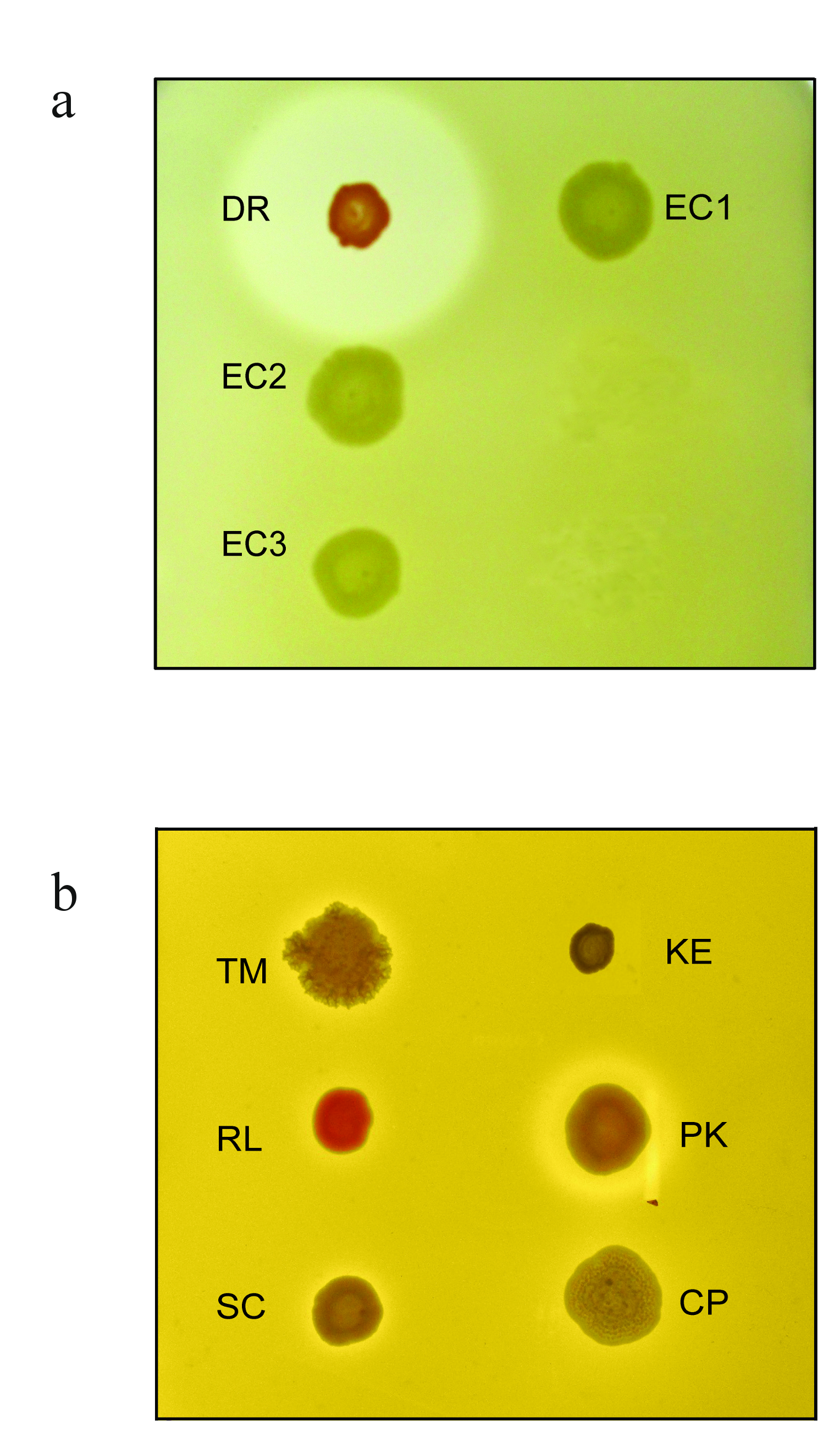

Supplement: S3 Fig — For bacteria the assay was performed on the beef agar, for fungi on YPD agar. Halos indicate the presence of active proteases. (TIF) [file pone.0189261.s004.tif]

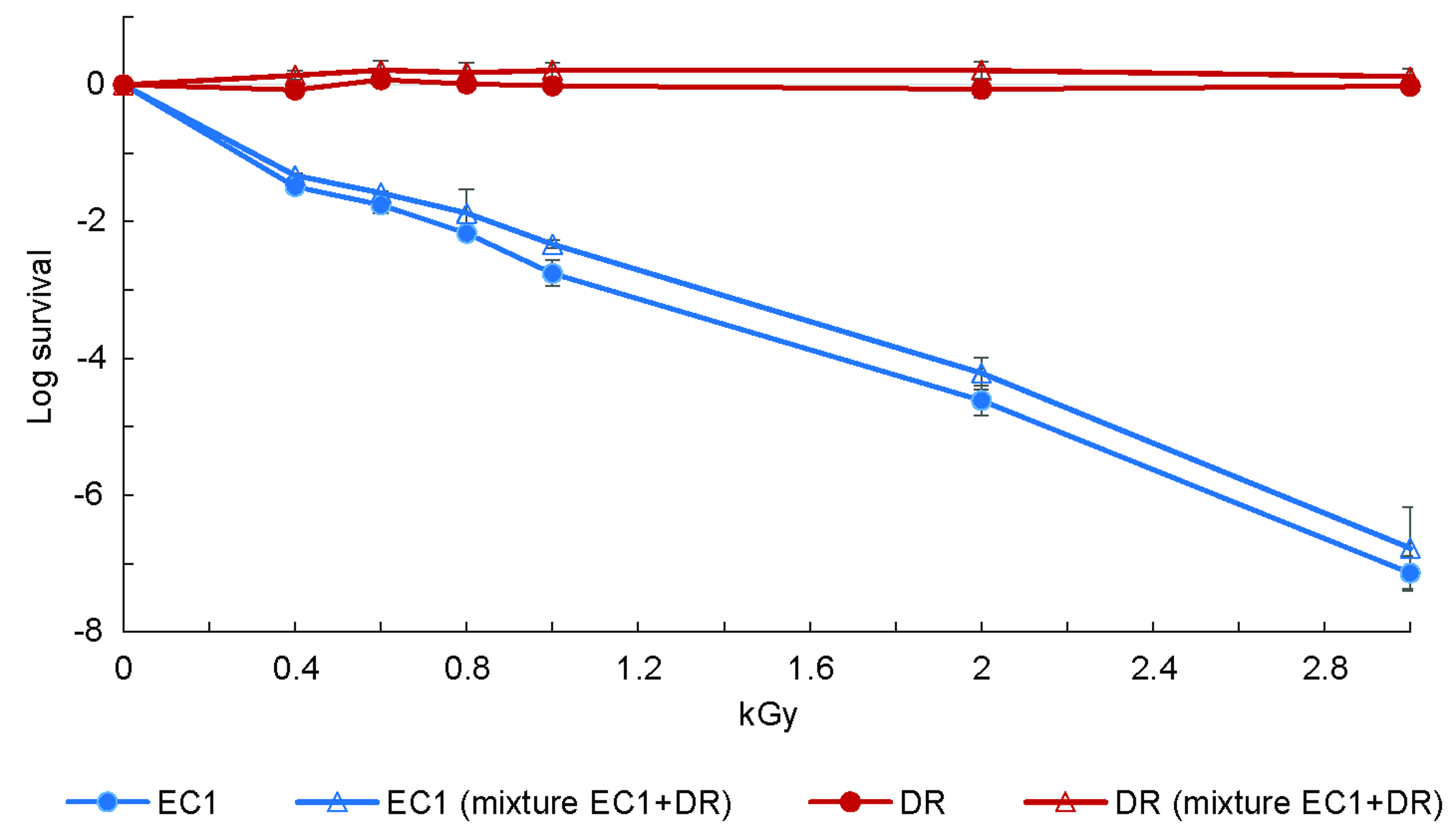

Supplement: S4 Fig — Blue circles: EC1 survival in pure culture; blue triangles: EC1 survival in mixed EC1+DR culture; red circles: DR survival in pure culture; red triangles: DR survival in mixed EC1+DR culture. (TIF) [file pone.0189261.s005.tif]
