# Supplementary material for: Microbial cells can cooperate to resist high-level chronic ionizing radiation
Source: PLoS One. 2017 Dec 20;12(12):e0189261. doi: 10.1371/journal.pone.0189261 (PMC5738026; doi:10.1371/journal.pone.0189261)
Supplement: S1 File — Contains the following supplementary tables: Table A. Sensitivity to AIR and CIR, measured by D10 and the ability to grow at 36 Gy/h, respectively, in 145 phylogenetically diverse fungi. Table B. Estimates of DNA DSB repair capabilities of the tested organisms. (DOCX) [file pone.0189261.s006.docx]

**Table A. Sensitivity to AIR and CIR, measured by D_10_ and the ability to grow at 36 Gy/h, respectively, in 145 phylogenetically diverse fungi.**

| **Strain number** | **Name** | **Phylum** | **D_10_, kGy** | **log_10_ [D_10_], kGy** | **Growth at 36 Gy/h** |
| --- | --- | --- | --- | --- | --- |
| EXF-7729 | *Cryptococcus laurentii* | Basidiomycota | 6.5 | 0.81 | 1 |
| EXF-3792 | *Cryptococcus fonsecae* | Basidiomycota | 4.2 | 0.62 | 1 |
| EXF-6424 | *Trichosporon moniliiforme* | Basidiomycota | 4.1 | 0.61 | 1 |
| EXF-6430 | *Ustilago davisii* | Basidiomycota | 4.0 | 0.60 | 1 |
| EXF-5822 | *Saccharomyces cerevisiae* | Ascomycota | 3.6 | 0.56 | 1 |
| EXF-6246 | *Saccharomyces cerevisiae* | Ascomycota | 3.5 | 0.54 | 0 |
| CBS 102277 | *Rhizopus rhizopodiformis* | Zygomycota | 3.5 | 0.54 | 1 |
| EXF-6761 | *Saccharomyces cerevisiae* | Ascomycota | 3.5 | 0.54 | 1 |
| EXF-6056 | *Calcarisporiella thermophila* | Ascomycota | 3.2 | 0.51 | 0 |
| EXF-5294 | *Saccharomyces cerevisiae* | Ascomycota | 3.2 | 0.51 | 0 |
| CBS 112285 | *Rhizopus microsporus* | Zygomycota | 3.2 | 0.51 | 1 |
| CBS 111.69 | *Corynascus sepedonium* | Ascomycota | 3.1 | 0.49 | 0 |
| EXF-4909 | *Saccharomyces bayanus* x *cerevisiae* | Ascomycota | 3.0 | 0.48 | 1 |
| EXF-5282 | *Saccharomyces cerevisiae* | Ascomycota | 3.0 | 0.48 | 1 |
| EXF-5284 | *Saccharomyces cerevisiae* | Ascomycota | 3.0 | 0.48 | 1 |
| EXF-6248 | *Saccharomyces cerevisiae* | Ascomycota | 3.0 | 0.48 | 0 |
| EXF-6408 | *Metschnikowia fructicola* | Ascomycota | 3.0 | 0.48 | 1 |
| EXF-5295 | *Saccharomyces cerevisiae* | Ascomycota | 2.8 | 0.45 | 1 |
| EXF-7173 | *Saccharomyces paradoxus* | Ascomycota | 2.8 | 0.45 | 0 |
| EXF-4916 | *Saccharomyces cerevisiae* | Ascomycota | 2.6 | 0.41 | 1 |
| EXF-5281 | *Saccharomyces cerevisiae* | Ascomycota | 2.6 | 0.41 | 1 |
| EXF-5735 | *Saccharomyces cerevisiae* | Ascomycota | 2.6 | 0.41 | 1 |
| EXF-5046 | *Saccharomyces cerevisiae* | Ascomycota | 2.5 | 0.40 | 1 |
| EXF-7135 | *Saccharomyces paradoxus* | Ascomycota | 2.5 | 0.40 | 1 |
| EXF-7200 | *Saccharomyces cerevisiae* | Ascomycota | 2.5 | 0.40 | 1 |
| EXF-8528 | *Rhodotorula nothofagi* | Basidiomycota | 2.5 | 0.40 | 0 |
| EXF-1920 | *Mucor* sp. | Zygomycota | 2.4 | 0.38 | 1 |
| EXF-6676 | *Saccharomyces paradoxus* | Ascomycota | 2.4 | 0.38 | 0 |
| EXF-8581 | *Rhodotorula fujisanensis* | Basidiomycota | 2.1 | 0.32 | 0 |
| EXF-308 | *Rhodotorula rubra* | Basidiomycota | 2.0 | 0.30 | 1 |
| EXF-3422 | *Saccharomyces cerevisiae* | Ascomycota | 2.0 | 0.30 | 1 |
| EXF-4911 | *Saccharomyces cerevisiae* | Ascomycota | 2.0 | 0.30 | 1 |
| EXF-5042 | *Saccharomyces cerevisiae* | Ascomycota | 2.0 | 0.30 | 1 |
| EXF-5248 | *Saccharomyces cerevisiae* | Ascomycota | 2.0 | 0.30 | 1 |
| EXF-5283 | *Metschnikowia pulcherrima* | Ascomycota | 2.0 | 0.30 | 1 |
| EXF-5293 | *Saccharomyces bayanus* | Ascomycota | 2.0 | 0.30 | 1 |
| EXF-5872 | *Saccharomyces cerevisiae* | Ascomycota | 2.0 | 0.30 | 0 |
| EXF-6247 | *Saccharomyces cerevisiae* | Ascomycota | 2.0 | 0.30 | 0 |
| EXF-6398 | *Pichia kudriavzevii* | Ascomycota | 2.0 | 0.30 | 1 |
| EXF-6684 | *Saccharomyces cerevisiae* | Ascomycota | 2.0 | 0.30 | 1 |
| EXF-7284 | *Saccharomyces kudriavzevii* | Ascomycota | 2.0 | 0.30 | 0 |
| EXF-5297 | *Saccharomyces cerevisiae* | Ascomycota | 1.8 | 0.26 | 1 |
| EXF-5875 | *Saccharomyces cerevisiae* | Ascomycota | 1.8 | 0.26 | 0 |
| EXF-6464 | *Debaryomyces hansenii* | Ascomycota | 1.8 | 0.26 | 1 |
| EXF-7137 | *Saccharomyces paradoxus* | Ascomycota | 1.8 | 0.26 | 0 |
| EXF-7197 | *Saccharomyces cerevisiae* | Ascomycota | 1.8 | 0.26 | 1 |
| EXF-1630 | *Rhodotorula mucilaginosa* | Basidiomycota | 1.6 | 0.20 | 1 |
| EXF-5043 | *Saccharomyces cerevisiae* | Ascomycota | 1.6 | 0.20 | 1 |
| EXF-6789 | *Saccharomyces cerevisiae* | Ascomycota | 1.6 | 0.20 | 1 |
| EXF-1612 | *Rhodosporidium lusitaniae* | Basidiomycota | 1.5 | 0.18 | 0 |
| EXF-7207 | *Saccharomyces kudriavzevii* | Ascomycota | 1.5 | 0.18 | 0 |
| EXF-7211 | *Saccharomyces kudriavzevii* | Ascomycota | 1.5 | 0.18 | 0 |
| EXF-7288 | *Saccharomyces kudriavzevii* | Ascomycota | 1.5 | 0.18 | 0 |
| EXF-3501 | *Rhodosporidium diobovatum* | Basidiomycota | 1.4 | 0.15 | 1 |
| EXF-6410 | *Pichia fermentans* | Ascomycota | 1.4 | 0.15 | 1 |
| EXF-3697 | *Rhodosporidium kratochvilovae* | Basidiomycota | 1.2 | 0.08 | 1 |
| EXF-6402 | *Kazachstania exigua* | Ascomycota | 1.2 | 0.08 | 1 |
| EXF-6835 | *Saccharomyces cerevisiae* | Ascomycota | 1.2 | 0.08 | 0 |
| EXF-7202 | *Saccharomyces cerevisiae* | Ascomycota | 1.2 | 0.08 | 1 |
| EXF-8527 | *Rhodotorula colostri* | Basidiomycota | 1.2 | 0.08 | 0 |
| EXF-512 | *Rhodosporidium sphaerocarpum* | Basidiomycota | 1.1 | 0.04 | 1 |
| EXF-1529 | *Rhodotorula minuta* | Basidiomycota | 1.1 | 0.04 | 1 |
| EXF-1534 | *Rhodotorula lysinophila* | Basidiomycota | 1.1 | 0.04 | 1 |
| EXF-5557 | *Rhodotorula slooffiae* | Basidiomycota | 1.1 | 0.04 | 1 |
| EXF-5870 | *Saccharomyces cerevisiae* | Ascomycota | 1.1 | 0.04 | 0 |
| EXF-6435 | *Rhodotorula glutinis* | Basidiomycota | 1.0 | 0.00 | 1 |
| EXF-513 | *Rhodosporidium babjevae* | Basidiomycota | 1.0 | 0.00 | 0 |
| EXF-518 | *Pichia guilliermondii* | Ascomycota | 1.0 | 0.00 | 1 |
| EXF-1496 | *Pichia guilliermondii* | Ascomycota | 1.0 | 0.00 | 1 |
| EXF-3409 | *Cryptococcus liquefaciens* | Basidiomycota | 1.0 | 0.00 | 1 |
| EXF-3661 | *Rhodosporidium lusitaniae* | Basidiomycota | 1.0 | 0.00 | 0 |
| EXF-5733 | *Saccharomyces cerevisiae* | Ascomycota | 1.0 | 0.00 | 0 |
| EXF-6094 | *Rhodotorula calyptogenae* | Basidiomycota | 1.0 | 0.00 | 1 |
| EXF-6421 | *Schwanniomyces pseudopolymorphus* | Ascomycota | 1.0 | 0.00 | 0 |
| EXF-6425 | *Rhodotorula glutinis* | Basidiomycota | 1.0 | 0.00 | 1 |
| EXF-6453 | *Cyberlindnera saturnus* | Ascomycota | 1.0 | 0.00 | 1 |
| EXF-6780 | *Saccharomyces cerevisiae* | Ascomycota | 1.0 | 0.00 | 1 |
| EXF-7163 | *Saccharomyces kudriavzevii* | Ascomycota | 1.0 | 0.00 | 0 |
| EXF-7210 | *Saccharomyces kudriavzevii* | Ascomycota | 1.0 | 0.00 | 0 |
| EXF-7282 | *Saccharomyces kudriavzevii* | Ascomycota | 1.0 | 0.00 | 0 |
| EXF-7289 | *Saccharomyces kudriavzevii* | Ascomycota | 1.0 | 0.00 | 0 |
| EXF-7977 | *Candida sake* | Ascomycota | 1.0 | 0.00 | 1 |
| EXF-9815 | *Rhodotorula aurantiaca* | Basidiomycota | 1.0 | 0.00 | 0 |
| CBS 171.62 | *Chrysosporium tropicum* | Ascomycota | 0.9 | -0.05 | 1 |
| CBS 728.84 | *Chaetomium senegalensis* | Ascomycota | 0.9 | -0.05 | 0 |
| EXF-3800 | *Rhodotorula benthica* | Basidiomycota | 0.9 | -0.05 | 0 |
| EXF-3909 | *Rhodotorula laryngis* | Basidiomycota | 0.9 | -0.05 | 0 |
| EXF-7964 | *Wickerhamomyces anomalus* | Ascomycota | 0.9 | -0.05 | 1 |
| MD1149 | *Rhodotorula taiwanensis* | Basidiomycota | 0.8 | -0.10 | 1 |
| EXF-5871 | *Saccharomyces cerevisiae* | Ascomycota | 0.8 | -0.10 | 1 |
| EXF-7107 | *Geotrichum* sp. | Ascomycota | 0.8 | -0.10 | 1 |
| EXF-6218 | *Saccharomyces cerevisiae* | Ascomycota | 0.8 | -0.10 | 0 |
| EXF-6219 | *Saccharomyces cerevisiae* | Ascomycota | 0.8 | -0.10 | 0 |
| CBS 134000 | *Arthrinium pterospermum* | Ascomycota | 0.7 | -0.15 | 0 |
| EXF-5288 | *Kluyveromyces marxianus* | Ascomycota | 0.6 | -0.22 | 1 |
| EXF-6436 | *Occultifur externus* | Basidiomycota | 0.6 | -0.22 | 0 |
| EXF-6054 | *Talaromyces thermophilus* | Ascomycota | 0.5 | -0.30 | 0 |
| EXF-6051 | *Chaetomium thermophilum* var. *dissitum* | Ascomycota | 0.5 | -0.30 | 0 |
| EXF-82 | *Aspergillus fumigatus* | Ascomycota | 0.5 | -0.30 | 0 |
| CBS 406.68 | *Hormoconis resinae* | Ascomycota | 0.5 | -0.30 | 0 |
| CBS 528.71 | *Dactylomyces thermophilus* | Ascomycota | 0.5 | -0.30 | 0 |
| EXF-3801 | *Rhodosporidium fluviale* | Basidiomycota | 0.5 | -0.30 | 0 |
| EXF-4431 | *Paecilomyces fulvus* | Ascomycota | 0.5 | -0.30 | 1 |
| EXF-6057 | *Malbranchea cinnamomea* | Ascomycota | 0.5 | -0.30 | 1 |
| EXF-6060 | *Thermoascus aurantiacus* | Ascomycota | 0.5 | -0.30 | 0 |
| EXF-6463 | *Candida pseudolambica* | Ascomycota | 0.5 | -0.30 | 1 |
| EXF-7145 | *Saccharomyces cerevisiae* | Ascomycota | 0.5 | -0.30 | 0 |
| EXF-7167 | *Saccharomyces paradoxus* | Ascomycota | 0.5 | -0.30 | 1 |
| ATCC 22073 | *Stilbella thermophile* | Ascomycota | 0.5 | -0.30 | 1 |
| ATCC 46858 | *Scytalidium indonesicum* | Ascomycota | 0.5 | -0.30 | 1 |
| CBS 102200 | *Thielavia terricola* | Ascomycota | 0.5 | -0.30 | 1 |
| ATCC 36347 | *Sporotrichum thermophilum* | Basidiomycota | 0.5 | -0.30 | 0 |
| EXF-6061 | *Thielavia terrestris* | Ascomycota | 0.5 | -0.30 | 0 |
| CBS 734.71 | *Acremonium thermophilum* | Ascomycota | 0.4 | -0.40 | 1 |
| EXF-271 | *Chaetomium globosum* | Ascomycota | 0.4 | -0.40 | 0 |
| CBS 187.54 | *Amorphotheca resinae* | Ascomycota | 0.4 | -0.40 | 0 |
| CBS 241.64 | *Thermomyces stellatus* | Ascomycota | 0.4 | -0.40 | 1 |
| EXF-296 | *Phanerochaete chrysosporium* | Basidiomycota | 0.4 | -0.40 | 1 |
| CBS 643.91 | *Remersonia thermophila* | Ascomycota | 0.4 | -0.40 | 0 |
| EXF-1760 | *Aspergillus niger* | Ascomycota | 0.4 | -0.40 | 1 |
| ATCC 56490 | *Thermoascus aegyptiacus* | Ascomycota | 0.4 | -0.40 | 1 |
| EXF-4062 | *Neosartorya fischeri* | Ascomycota | 0.3 | -0.52 | 0 |
| EXF-6055 | *Humicola grisea var. thermoidea* | Ascomycota | 0.3 | -0.52 | 0 |
| CBS 281.67 | *Thermomyces ibadanensis* | Ascomycota | 0.3 | -0.52 | 0 |
| CBS 405.69 | *Corynascus thermophiles* | Ascomycota | 0.3 | -0.52 | 0 |
| CBS 597.83 | *Myceliophthora hinnulea* | Ascomycota | 0.3 | -0.52 | 0 |
| EXF-4434 | *Rhizomucor pusillus* | Zygomycota | 0.3 | -0.52 | 1 |
| EXF-6058 | *Paecilomyces formosa* | Ascomycota | 0.3 | -0.52 | 1 |
| EXF-6059 | *Thermomyces lanuginosus* | Ascomycota | 0.3 | -0.52 | 0 |
| ATCC 28236 | *Thielavia australiensis* | Ascomycota | 0.3 | -0.52 | 0 |
| CBS 100279 | *Talaromyces emersonii* | Ascomycota | 0.3 | -0.52 | 0 |
| EXF-301 | *Rhizomucor miehei* | Zygomycota | 0.3 | -0.52 | 0 |
| EXF-6062 | *Thermoascus thermophilus* | Ascomycota | 0.3 | -0.52 | 1 |
| CBS 179.67 | *Chaetomium thermophilum* v. *coprophile* | Ascomycota | 0.3 | -0.52 | 0 |
| CBS 181.67 | *Thermoascus crustaceus* | Ascomycota | 0.3 | -0.52 | 0 |
| CBS 406.69 | *Myceliophthora fergusii* | Ascomycota | 0.3 | -0.52 | 0 |
| CBS 413.71 | *Paecilomyces byssochlamydoides* | Ascomycota | 0.3 | -0.52 | 0 |
| EXF-589 | *Debaryomyces hansenii* | Ascomycota | 0.3 | -0.52 | 0 |
| CBS 886.97 | *Melanocarpus thermophiles* | Ascomycota | 0.3 | -0.52 | 0 |
| EXF-6063 | *Nodulisporium cylindroconium* | Ascomycota | 0.2 | -0.70 | 1 |
| EXF-2416 | *Aspergillus flavus* | Ascomycota | 0.2 | -0.70 | 0 |
| EXF-6050 | *Myceliophthora thermophila* | Ascomycota | 0.2 | -0.70 | 0 |
| EXF-5576 | *Exophiala dermatitidis* A | Ascomycota | 0.1 | -1.00 | 1 |
| EXF-5585 | *Exophiala dermatitidis* C | Ascomycota | 0.1 | -1.00 | 1 |
| EXF-5586 | *Exophiala dermatitidis* B | Ascomycota | 0.1 | -1.00 | 1 |

**Table B. Estimates of DNA DSB repair capabilities of the tested organisms.**

| **Species (abbreviation), kingdom** | **Strain** | **Observed growth-inhibitory critical dose rate (Gy/h) at high cell concentrations: lower and upper limits, mean estimate** | | | **D_10_ (kGy)** | **Haploid genome size (Mbp)** | **Predic-ted DSBs per genome at D_10_^‡^** | **Predicted DSBs per genome per hour at mean critical dose rate estimate** | **Reference for genome size** |
| --- | --- | --- | --- | --- | --- | --- | --- | --- | --- |
| *Trichosporon mucoides* (TM), fungi | EXF-1444 | 67 | 94 | 80.5 | 4.5 | 19.8 | 356.4 | 6.38 | ** [1] |
| *Rhodotorula lysinophila* (RL), fungi | EXF-1534 | 36 | 67 | 51.5 | 1.1 | 20.01 | 88.0 | 4.12 | ** [2] |
| *Pichia kudriavzevii* (PK), fungi | EXF-6398 | 36 | 67 | 51.5 | 2 | 12.94 | 103.5 | 2.67 | * [3] |
| *Saccharomyces cerevisiae* (SC), fungi | EXF-5294 | 36 | 67 | 51.5 | 3.2 | 12 | 153.6 | 2.47 | * [4] |
| *E. coli* (EC2), bacteria | CB1000 | 94 | 126 | 110 | 0.2 | 4.64 | 3.7 | 2.04 | * [5,6] |
| *D. radiodurans* (DR), bacteria | ATCC BAA-816 | 126 | 180 | 153 | 12 | 3.28 | 157.4 | 2.01 | [7] |
| *Kazachstania exigua* (KE), fungi | EXF-6402 | 13 | 36 | 24.5 | 1.2 | 18 | 86.4 | 1.76 | ** [8] |
| *E. coli* (EC3), bacteria | CB2000 | 67 | 94 | 80.5 | 0.25 | 4.64 | 4.6 | 1.49 | * [5,6] |
| *Candida parapsilosis*, (CP), fungi | EXF-517 | 13 | 36 | 24.5 | 1 | 13.1 | 52.4 | 1.28 | * [9] |
| *E. coli* (EC1), bacteria | K-12 MG1655 CF1648 | 36 | 67 | 51.5 | 0.25 | 4.64 | 4.6 | 0.96 | [5,6] |

**^‡^**The DSB yield was assumed to be 0.004 DSB/(Gy×Mbp) for all organisms [10,11]. Organism-specific values may differ up to several-fold from this average value. Lower and upper bounds for growth-inhibitory critical dose rate refer to CIR dose rates between which the organism’s response changed from visible growth to complete inhibition of growth under aerobic conditions at the maximum tested cell concentration. D_10_ refers to the dose of AIR which reduced clonogenic survival by 10-fold. The organisms are ranked in order of decreasing DSBs per genome per hour at critical dose rate. * indicates same species, different strain; ** indicates same genus, different species.

**References**

1. Kourist R, Bracharz F, Lorenzen J, Kracht ON, Chovatia M, Daum C, et al. Genomics and Transcriptomics Analyses of the Oil-Accumulating Basidiomycete Yeast Trichosporon oleaginosus: Insights into Substrate Utilization and Alternative Evolutionary Trajectories of Fungal Mating Systems. MBio. 2015;6: e00918. doi:10.1128/mBio.00918-15

2. Firrincieli A, Otillar R, Salamov A, Schmutz J, Khan Z, Redman RS, et al. Genome sequence of the plant growth promoting endophytic yeast Rhodotorula graminis WP1. Front Microbiol. 2015;6: 978. doi:10.3389/fmicb.2015.00978

3. Chan GF, Gan HM, Ling HL, Rashid NAA. Genome sequence of Pichia kudriavzevii M12, a potential producer of bioethanol and phytase. Eukaryot Cell. 2012;11: 1300–1. doi:10.1128/EC.00229-12

4. Sharma A, Gaidamakova EK, Grichenko O, Matrosova VY, Hoeke V, Klimenkova P, et al. Across the tree of life, radiation resistance is governed by antioxidant Mn(2+), gauged by paramagnetic resonance. Proc Natl Acad Sci U S A. National Academy of Sciences; 2017; 201713608. doi:10.1073/pnas.1713608114

5. Harris DR, Pollock S V, Wood EA, Goiffon RJ, Klingele AJ, Cabot EL, et al. Directed evolution of ionizing radiation resistance in Escherichia coli. J Bacteriol. 2009;191: 5240–52. doi:10.1128/JB.00502-09

6. Krisko A, Radman M. Protein damage and death by radiation in Escherichia coli and Deinococcus radiodurans. Proc Natl Acad Sci U S A. 2010;107: 14373–7. doi:10.1073/pnas.1009312107

7. Lin J, Qi R, Aston C, Jing J, Anantharaman TS, Mishra B, et al. Whole-genome shotgun optical mapping of Deinococcus radiodurans. Science. 1999;285: 1558–62. Available: http://www.ncbi.nlm.nih.gov/pubmed/10477518

8. Safar SVB, Gomes FCO, Marques AR, Lachance M-A, Rosa CA. Kazachstania rupicola sp. nov., a yeast species isolated from water tanks of a bromeliad in Brazil. Int J Syst Evol Microbiol. 2013;63: 1165–8. doi:10.1099/ijs.0.048462-0

9. Maguire SL, ÓhÉigeartaigh SS, Byrne KP, Schröder MS, O’Gaora P, Wolfe KH, et al. Comparative genome analysis and gene finding in Candida species using CGOB. Mol Biol Evol. 2013;30: 1281–91. doi:10.1093/molbev/mst042

10. Makarova KS, Omelchenko M V, Gaidamakova EK, Matrosova VY, Vasilenko A, Zhai M, et al. Deinococcus geothermalis: the pool of extreme radiation resistance genes shrinks. PLoS One. 2007;2: e955. doi:10.1371/journal.pone.0000955

11. Daly MJ. Death by protein damage in irradiated cells. DNA Repair (Amst). 2012;11: 12–21. doi:10.1016/j.dnarep.2011.10.024
